# Analysis of p53-Independent Functions of the Mdm2-MdmX Complex Using Data-Independent Acquisition-Based Profiling

**DOI:** 10.3390/proteomes13020018

**Published:** 2025-05-22

**Authors:** Anu Jain, Rafaela Muniz de Queiroz, Jayanta K. Chakrabarty, Karl A. T. Makepeace, Carol Prives, Lewis M. Brown

**Affiliations:** 1 Department of Biological Sciences, Columbia University, New York, NY 10027, USA; 2 Quantitative Proteomics and Metabolomics Center, Columbia University, New York, NY 10027, USA

**Keywords:** data-independent acquisition, label-free proteomics, Mdm2, MdmX, p53-independent functions, cancer

## Abstract

Background: We utilized data-independent acquisition (DIA) to study the poorly understood biology of Mdm2 and MdmX in a p53-null context. Mdm2 and MdmX form an E3-ligase complex that has as its most well-studied function the negative regulation of the tumor suppressor p53; however, it is also known to interact with many other proteins in a p53-independent manner. Methods: In this work, small-molecule and siRNA-based technology were used to modify Mdm2/MdmX activity in a human non-small-cell lung carcinoma cell line lacking p53 expression. Study of the proteome of these cells helped identify biological processes where Mdm2 and MdmX may play roles in a p53-independent manner. Proteins from H1299 cells, treated with the drug MEL23 or siRNA against Mdm2 or MdmX, were analyzed. Results: Protein ontology and function were analyzed, revealing which pathways are affected by modulation of the proteins that form the complex. Insights into how those functions are dependent on the activity of the complex also gained via comparisons among the three groups of samples. Conclusions: We selected a potential target from the DIA analysis and validated it by immunoblotting and qPCR, and this allows us to demonstrate a new interaction partner of the Mdm2-MdmX complex in human cells.

## 1. Introduction

The tumor suppressor p53, also known as the guardian of the genome, is a transcription factor activated in response to various cellular stresses, for instance, DNA damage, metabolic stress, hypoxia, and oxidative stress, among others. It activates/suppresses sets of target genes, leading to a cascade of cellular events including, but not limited to, DNA repair, cell cycle arrest, and apoptosis [1,2,3,4,5,6]. Mdm2 (also known as Hdm2 in humans) and its homolog MdmX (also known as Mdm4 or HdmX/Hdm4 in humans) form an E3 ligase complex, which is the main regulator of p53 protein levels in cells [7,8,9,10,11], but also are shown to regulate different proteins and cellular processes [12]. p53 function, however, is usually lost in cancers due to mutations in the gene [13], while Mdm2 and MdmX are found to be amplified in some tumor types [14]. Thus, we are interested in understanding the role of Mdm2 and MdmX in cancer cells that have lost p53 expression, to further elucidate p53-independent functions of this protein complex.

In this study, we aimed to elucidate p53-independent roles of Mdm2 and MdmX using a high-resolution mass spectrometry with a DIA method using PEAKS Studio software. For this work, we used H1299 cells, a human non-small-cell lung carcinoma cell line which has a homozygous partial deletion of p53 and does not express p53. We treated H1299 cells with MEL23 (Mdm2 E3 ligase inhibitor) [15], siRNA against Mdm2, or siRNA against MdmX. MEL23 affects the ubiquitin ligase activity of the Mdm2-MdmX complex without decreasing protein expression, and siRNA affects the transcript level with consequent downregulation of protein levels.

In contrast to DIA, data-dependent acquisition (DDA) is the more conventional method used previously in this field for global proteomic profiling, in which all the ions are scanned at the MS1 stage and then a few selected ions are fragmented at the MS2 stage, allowing the identification of the selected ions. The ion selection for fragmentation is stochastic and usually decided by abundance. This strategy results in variability in the data, and due to the selection of fewer ions, less coverage is achieved, which can further be decreased depending on the complexity or dynamic range of the sample type being analyzed. In contrast, in DIA, the method that we selected, every precursor ion in a set m/z window is fragmented and identified, which lowers the variability and increases the coverage. DIA has evolved as a highly reproducible approach for proteomic profiling. In DDA, only selected precursor ions are fragmented. Given its reported reproducibility, higher accuracy, and sensitivity, the DIA method is gradually becoming the method of preference over the conventional DDA method [16,17,18,19]. For this work, we implemented the DIA method, employing a widely used and well-validated software package, PEAKS Studio (Bioinformatics Solutions Inc., Waterloo, Ontario, Canada), which has been shown to be effective in a wide variety of proteomics applications [20,21,22,23,24,25,26,27,28,29], including work relating to cancer [30,31,32,33,34,35,36,37]. The extensive literature cited supporting this tool typically employed label-free quantitation (LFQ). This powerful tool integrates de novo sequencing with database searching. DIA analysis with library searches in PEAKS has been shown to be effective, reliably detecting 5–30% more peptide precursors compared to other DIA software [38]. PEAKS has also been used effectively in other DIA protein analysis studies [39], including library-based work [40,41], and for library-based DIA peptide detection [42,43]. While there is important emerging literature on this topic, more studies such as the work described herein are necessary, given the increasing importance of DIA in proteomics.

A spectral library generated with information from DDA can be used to analyze the DIA data [17]. Here, we used a high-resolution Orbitrap mass spectrometer to acquire data and used PEAKS Studio (V. 10.6 Bioinformatics Solutions Inc., Waterloo, ON, Canada) to generate spectral libraries and analyze the DIA data [38].

## 2. Materials and Methods

### 2.1. Chemicals and Reagents

All chemicals were reagent-grade. All solvents, including water, used in proteomics sample preparation and chromatography, were Optima LC/MS-grade (ThermoFisher Scientific, Fair Lawn, NJ, USA).

### 2.2. Study Design

**Figure 1:**
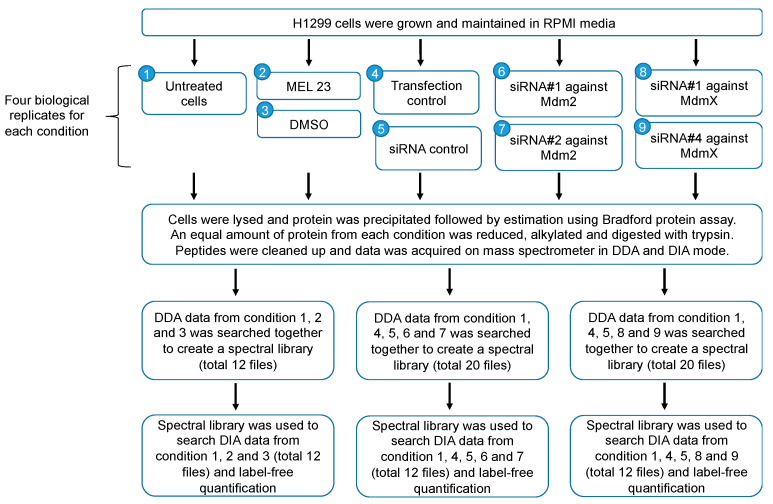
Schematic diagram depicting the study design.

Human lung adenocarcinoma cell line H1299 was treated with either MEL23 or DMSO (vehicle control). In the siRNA group, cells were treated with a transfection reagent only, control siRNA, siRNA against Mdm2 (two different siRNAs were used against Mdm2; siRNA1 and siRNA2), or siRNA against MdmX (two different siRNAs were used against MdmX; siRNA1 and siRNA4). A total of nine different conditions were analyzed (Figure 1).

The condition named “transfection control” comprises cells transfected using a transfection reagent only, with the addition of no siRNA. “Untreated” conditions are cells exposed only to media. Each treatment was carried out in four different biological replicates.

### 2.3. Cell Culture

The human lung adenocarcinoma line H1299 was purchased from the American Type Culture Collection (ATCC) (#CRL-5803). H1299 cells were grown in RPMI 1640 medium (#11875119, Thermo Scientific, Carlsbad, CA, USA) supplemented with 10% heat-inactivated fetal bovine serum (FBS) (#900108H, Gemini, West Sacramento, CA, USA), 100 units of penicillin, and 100 mg/mL streptomycin (#15140122, Thermo Scientific). Cells were maintained at 37 °C with 5% CO_2_ and were subcultured using trypsin-EDTA (#15090-046, Gibco, Grand Island, NY, USA) every 2 or 3 days. Before samples were collected, the cell line was tested for its mycoplasma contamination using the LookOut Mycoplasma PCR detection kit (#MP0035, Sigma-Aldrich, St. Louis, MO, USA) and found to be free of contamination.

### 2.4. siRNA Transfection and Drug Treatment

Cells were seeded in 6-well plates. The next day, cells in the siRNA group were transfected with siRNAs against Mdm2 (Ambion, Silencer Select s8630 (siRNA1) and s224037 (siRNA2)) or MdmX (Qiagen, FlexiTubes SI00037163 (siRNA1) and Sigma-Aldrich costume siRNA (siRNA4), sequence: 5′-AGAUUCAGCUGGUUAUUAA-3′) or with an siRNA negative control (Ambion, Silencer Select #4390843), using Lipofectamine RNAiMax transfection reagent (#13778150, Thermo Scientific). Cells were treated with siRNA for 48 h. Cells in the drug group were treated with MEL23 (7 µM) (373227, Sigma-Aldrich) or DMSO (vehicle). After 24 h, cells were lysed in lysis buffer (0.3% SDS, 6 mM DTT in 1X TBS) and protein was precipitated. Each experiment was repeated four times to generate biological replicates.

### 2.5. Sample Preparation for Proteomic Analysis, Mass Spectrometry, and Data Analysis

Protein was extracted from the cell lysates and digested and peptides were prepared for mass spectrometry with methods similar to those described previously [44]. Specifically, protein was extracted from the cell lysates precipitated with chloroform-methanol as described previously [44] and dissolved in 30 µL of solubilization buffer (100 mM ammonium bicarbonate, 8 M urea, 0.1 M DTT). The protein concentration was measured with the Bradford reagent and an equal amount of protein (~30 µg) from each sample was processed.

Cysteine residues were reduced with DTT (2.5 mM) and alkylated with iodoacetamide (15 mM) with 40 min of incubation at room temperature after each addition. Sequencing-grade trypsin (Promega, #V5111, Madison, WI, USA) was added (protein: trypsin~50:1) and incubated overnight at 37 °C. Following digestion, samples were acidified to pH < 2 with TFA, kept on ice for 5 min to precipitate lipids, and centrifuged for 15 min at 20,000× *g*. Samples were desalted using Nestgroup C18 Macrospin columns (#SMM SS18V) following the manufacturer’s protocol. Briefly, columns were conditioned with 100% ACN and flushed twice with Optima water. Peptides were loaded onto the column followed by washing twice with 0.1% TFA. Peptides were eluted with 80% ACN and 0.1% formic acid in water. Eluted peptides were lyophilized, and redissolved in 3% ACN and 0.1% formic acid.

### 2.6. Mass Spectral Libraries

For mass spectral libraries, spectra for all samples were acquired on a Q Exactive HF (Orbitrap) mass spectrometer (ThermoFisher Scientific, Bremen, Germany) in the positive ion mode using the DDA method as described previously [45]. Peptides were loaded on a 75 μm ID × 2 cm Acclaim PepMap C18 trap column prior to separation at 5 μL/min. As described previously, separation was performed with a 75 μm ID × 50 cm Acclaim PepMap C18 column on an Ultimate 3000 RSLCnano liquid chromatograph (ThermoFisher Scientific, Sunnyvale, CA, USA) [45].

The MS1 acquisition method included a resolution of 120,000, AGC target of 3 × 10^6^, and injection time of 30 ms with a scan range of 400 to 2000 m/z. For MS2 acquisition, the resolution was set to 15,000 with an ion injection time of 100 ms, AGC target of 1 × 10^5^, and isolation window of 1.2 m/z. The top 15 precursor ions were selected for MS2 fragmentation. DDA data for library construction were analyzed with PEAKS Studio (V. 10.6 Bioinformatics Solutions Inc., Waterloo, ON, Canada) and searched against the Human UniProt database (Reference Proteome: UP000005640; Release # 2020_04; 42,347 sequences with contaminants). Search parameters included fixed modification on cysteine (carbamidomethyl) and a mass error tolerance of 10 ppm for MS and 0.02 Da for MS/MS. Variable modifications were oxidation (methionine) and acetylation (protein N-term). For HeLa digest, the ADH1 sequence was added to the Human UniProt database.

Data obtained from all 36 samples were divided into 3 different projects for searches in PEAKS Studio (Figure 1). Data from untreated cells DMSO- and MEL23-treated cells were searched together in one project. In the other two projects, data from untreated cells, transfection control cells, siRNA negative control-treated cells, and Mdm2 siRNA- or MdmX siRNA-treated cells were searched. Search parameters were the same for all three projects. Spectral libraries were built with the database search results of each search and used to analyze the respective DIA results.

### 2.7. Data-Independent Acquisition

For the acquisition of data in the DIA mode, peptides were loaded and separated on an Ultimate 3000 RSLCnano liquid chromatograph following a similar method as for the DDA acquisition described above. The mass spectrometer method included a resolution of 120,000 with an ion injection time of 20 ms and an AGC target of 3 × 10^6^ for a scan range of 350–1200 m/z at the MS1 level. For MS2, overlapping isolation windows of 30 m/z were set at a resolution of 30,000.

### 2.8. Mass Spectrometry Data Analysis

A spectral library search with PEAKS Studio (V. 10.6 Bioinformatics Solutions Inc., Waterloo, On, Canada), using the libraries built as described above, was performed to analyze the DIA data. Similar to the searches conducted to build spectral libraries, DIA data searches were performed in three different projects, as explained above (Figure 1).

Label-free quantification was performed at the MS1 peptide abundance and rolled up to the protein abundance by the software. The sum of the top three peptide precursor areas was used for protein quantification in PEAKS. The ANOVA (Welch) method was used to calculate the significance and generate a *p*-value for each protein.

To test the workflow and guard against spurious false positives, a null trial was performed where a HeLa digest (Pierce Protein digest #88328) was divided in half and 250 ng was loaded on-column in triplicate from each half and analyzed with DIA using a DDA library in the same fashion as for the H1299 cell digests.

All mass spectrometry raw data have been deposited in an international repository (5 May 2025) (MassIVE at https://massive.ucsd.edu) under accession #MSV000093577.

### 2.9. Post Hoc Statistical Analysis

Statistical processing was conducted in R (V 4.1.2) using the package rstatix (V 0.7.0). Protein abundance values were log 10 transformed to generate post hoc *p*-values using the Games–Howell method. MEL23-treated cells were compared to DMSO-treated or untreated cells, and siRNA-treated cells were compared to untreated cells, transfection controls, or control siRNA-treated cells. For differential abundance, proteins identified with ≥2 peptides, ≥1.5 or ≤0.667 fold-change, and an ANOVA post hoc *p*-value < 0.05 were included. In the drug treatment group, proteins dysregulated in the MEL23-treated cells compared to DMSO-treated or untreated cells were considered. In the siRNA treatment group, proteins dysregulated in either of the two siRNA-treated cells for each protein (Mdm2 and MdmX) compared to control siRNA-treated cells were considered.

### 2.10. Gene Ontology and Biological Inference

Gene ontology and pathway enrichment analysis were carried out to infer the biological significance of the differentially abundant proteins. Fisher’s exact test using the PANTHER [46] overrepresentation test was used for gene ontology enrichment analysis. DAVID (Database for Annotation, Visualization, and Integrated Discovery) [47,48] was used to detect the enriched pathways.

### 2.11. Immunoblotting

Aliquots of the same samples were used for mass spectrometry as well as blots. Laemmli buffer was added to the samples used for the MS run, followed by a 2 min heat step at 95 °C. Samples were loaded in equal protein amounts and fractionated by SDS-PAGE. Proteins were transferred to a PVDF membrane (IPVH00010, Immobilon Millipore, Burlington, MA, USA) and blocked for 1 h using Tris-buffered saline–0.05 % Tween 20 with 3% milk. Membranes were incubated overnight at 4 °C with primary antibody (Cell Signaling Technology, MDM2 (D1V2Z) Rabbit mAb #86934; Sigma-Aldrich, Anti-β-actin #A2228; Cell Signaling Technology, Anti-HMGCS1 (HMCS1_HUMAN) antibody #ab155787; Abcam, Anti-MdmX mAb 8C6 was produced at Dr. Jiandong Chen’s lab (Moffitt Cancer Center & Research Institute, Tampa, FL, USA) and gifted to our group), washed with Tris-buffered saline–0.05% Tween 20 and incubated with secondary HRP-conjugated antibody (Sigma-Aldrich, anti-mouse peroxidase #A4416; Sigma-Aldrich, anti-rabbit peroxidase #A6154) for 1 h. After washes, blots were developed by chemiluminescence detection using chemiluminescent horseradish peroxidase reagent (EMD Millipore, Immobilon, cat# WBKLS0050) according to the manufacturer’s instructions.

### 2.12. Co-Immunoprecipitation

Co-immunoprecipitation was performed by harvesting cells after treatment with MG132 for 4 h, to prevent degradation of possible partners, followed by lysing samples in NP-40 lysis buffer. Lysates containing 1 mg of protein were incubated overnight with primary antibody mix against Mdm2 or IgG control antibody at 4 °C with constant rotation, followed by 1 h incubation with Protein G agarose pre-blocked beads (Millipore, catalog no. 16-266, Burlington, MA, USA) at 4 °C with constant rotation. Beads were washed 4 times with lysis buffer or PBS; then, protein was eluted from beads with Laemmli buffer and boiled for 5 min. Samples were analyzed by immunoblotting.

### 2.13. Quantitative PCR

RNA was isolated from cells transfected with siRNAs against Mdm2 or siRNA control and harvested after 24 h. To isolate RNA, we used a Qiagen RNeasy kit (Germantown, MD, USA) according to the manufacturer’s instructions. cDNA was generated from isolated RNA using a Qiagen Quantitect reverse transcription kit (Germantown, MD, USA). The cDNA products were analyzed by qPCR using SYBR Green dye (ThermoFisher Scientificm Carlsbad, CA, USA) according to the manufacturer’s instructions. Quantitative PCR analysis was performed in an AB StepOnePlus real time PCR detection system (Thermo Fisher Scientific, Carlsbad, CA, USA) with the following protocol: polymerase activation and DNA denaturation for 30 s at 95 °C; amplification denaturation for 5 s at 95 °C and annealing for 30 s at 62 °C with 40 cycles; and melt curves generated at 65–95 °C with 0.5 °C increments (5 s/step). Quantification cycle (Cq) values were recorded by StepOne software. Relative changes in cDNA levels were calculated using the comparative Ct method (ΔΔCT method). RPL32 was used as the housekeeping gene.

## 3. Results

### 3.1. Biological Proteomics Results: H1299 Cells with Modulated Mdm2/MdmX Expression or Activity

**Figure 2:**
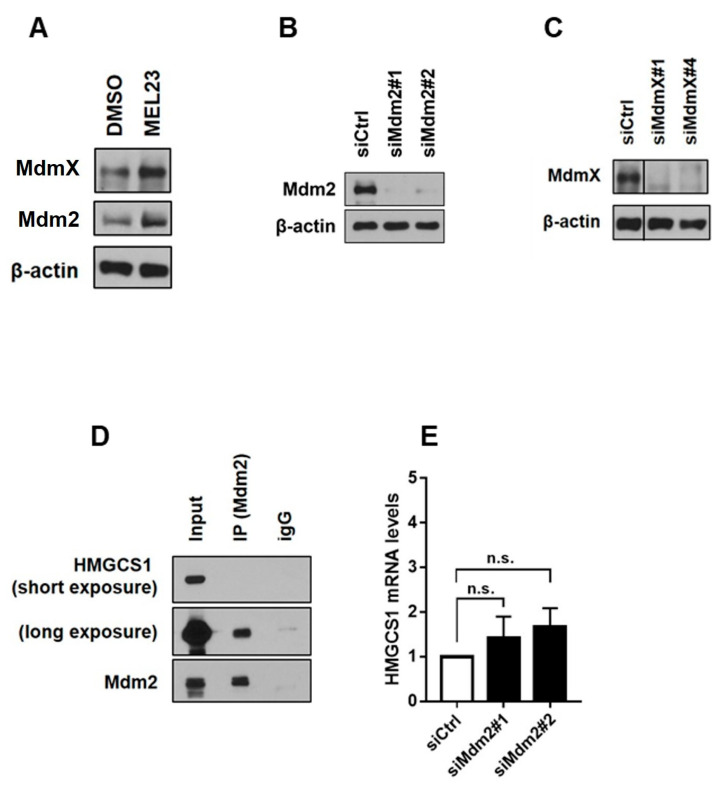
Validation of protein levels by immunoblots and RNA levels by quantitative PCR. (**A**) Validation of MEL23 treatment, with protein levels of Mdm2 and MdmX, both targets of the Mdm2/X complex activity, after 24 h treatment with 7 µM of MEL23 or vehicle control (DMSO), analyzed by immunoblotting. (**B**,**C**) Mdm2 and MdmX silencing, with protein levels measured by immunoblot for Mdm2 and MdmX after transfection using siRNAs against Mdm2 or MdmX. (**D**) Co-immunoprecipitation of HMCS1_HUMAN (HMGCS1) and Mdm2. Mdm2 was immunoprecipitated in H1299 whole-cell lysate, and the sample was analyzed by immunoblotting for the presence of HMCS1_HUMAN. IgG was used as an immunoprecipitation control. (**E**) mRNA levels of HMGCS1 in Mdm2-silenced cells as measured by quantitative PCR (n.s.-non-significant). See Figure S7 for original blot images and Table S6 for corresponding densitometry measurements.

In order to understand the p53-independent roles of the Mdm2-MdmX complex in cancer cells, we used three approaches: the knockdown of each component of the complex, using two different siRNAs for Mdm2 and two different siRNAs against MdmX, as well as the siRNA scramble used as a control, and a pharmacological strategy involving treatment with the inhibitor of the Mdm2/MdmX E3-ligase activity, MEL23, or DMSO as a vehicle control. The same methods used for the HeLa proteome were used for H1299 samples to identify and quantify proteins across whole-cell lysates of 36 separate cell cultures (four biological replicates for nine different groups and three treatments), as described in Figure 1. The confirmation of the knockdown of Mdm2 and MdmX, as well as the confirmation of the drug activity (seen by the accumulation of Mdm2 and MdmX, targets of the Mdm2-MdmX complex, in response to the drug), were shown in immunoblot of cells collected and processed for mass spectrometry (Figure 2A–C).

**Figure 3:**
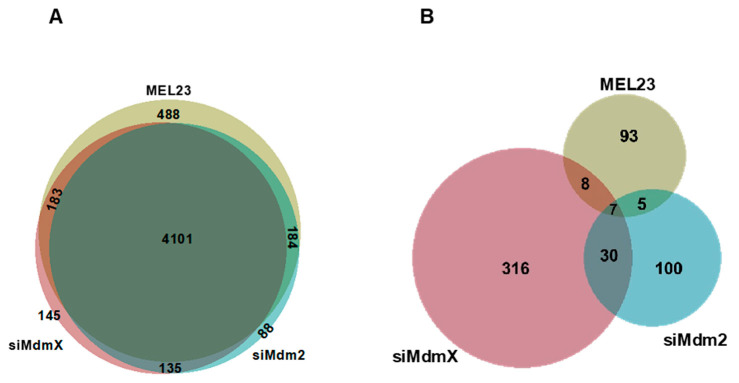
Protein identification in each treatment group. Venn diagram representing the number of proteins identified with ≥2 peptides, including isoforms, in each treatment group. (**A**) The total number of proteins identified. (**B**) The number of proteins differentially abundant in each treatment. For differential abundance, proteins identified with ≥2 peptides, ≥1.5 or ≤0.667 fold-change and an ANOVA *p*-value < 0.05 were considered. In the drug treatment group, proteins dysregulated in the MEL23-treated cells compared to DMSO-treated or untreated cells were included. In the siRNA treatment group, proteins dysregulated in either of the two siRNA treatments for each protein (Mdm2 and MdmX) compared to control siRNA-treated cells were included.

In the MEL23-treated group, 4956 proteins with ≥2 peptides (including isoforms) were identified. With the siRNA treatment against Mdm2 and MdmX, 4508 and 4564 proteins with ≥2 peptides (including isoforms) were identified, respectively. Among the three treatments, 4101 proteins were identified in common (Figure 3A, Tables S1–S3).

We observed that some of the dysregulated proteins were found in common in all three treatment types or some were exclusive to each treatment type. We also found an overlap among the dysregulated proteins between the two treatment types (MEL23 and siRNA against Mdm2, MEL23 and siRNA against MdmX, siRNA against Mdm2 and MdmX) (Figure 3B).

Some of the upregulated proteins identified upon MEL23 treatment (Table S1) included transcription factor Jun (JUN_HUMAN, a transcription factor [49]), cell division cycle protein 123 homolog (CD123_HUMAN, cell cycle regulatory protein), replication stress response regulator SDE2 (SDE2_HUMAN, cell cycle regulatory protein [50]), and TNF receptor-associated factor 1 (TRAF1_HUMAN, regulator of NF-kappaβ and JNK pathway [51]). Proteins found to be downregulated (Table S1) included stearoyl-CoA desaturase (SCD_HUMAN, a protein linked to fatty acid metabolism and ferroptosis [52,53]). Some of the proteins were found to be dysregulated only upon MEL23 treatment, including RNA binding protein 38 (RBM38_HUMAN, upregulated, involved in cell cycle regulation [54]), RNA binding protein 24 (RBM24_HUMAN, upregulated, involved in pre-mRNA splicing, stability, and cell differentiation [55,56]), receptor-type tyrosine-protein phosphatase F (PTPRF_HUMAN, upregulated, cell adhesion receptor), DNA repair protein RAD51 homolog 1 (RAD51_HUMAN, upregulated, involved in homologous recombination DNA repair pathway [57]), and phospholipid hydroperoxide glutathione peroxidase (GPX4_HUMAN, downregulated, key regulatory protein of ferroptosis [58,59]), suggesting that these proteins might be affected by the E3 ligase activity of the Mdm2-MdmX complex and not by the individual proteins of the complex, as they are affected only upon disruption of E3 ligase activity of the Mdm2-MdmX complex.

Downregulation of Mdm2 levels affected the abundance of a number of proteins (Table S2), including Ras-related protein Rap-2c (RAP2C_HUMAN, upregulated, a GTP binding protein [60]), nucleus accumbens-associated protein 1 (NACC1_HUMAN, upregulated, a transcription repressor involved in tumor progression [61]), vigilin (VIGLN_HUMAN, downregulated, plays a role in sterol metabolism), and ADP-ribosylation factor GTPase-activating protein 3 (ARFG3_HUMAN, downregulated, a GTPase-activating protein [62]). Interestingly, only one protein, PRA1 family protein 3 (PRAF3_HUMAN), was found to be upregulated only in the siRNA treatment against Mdm2, which regulates the intracellular concentrations of glutamate (Table S2).

Proteins found to be dysregulated upon MdmX modification (Table S3) included transcription elongation factor A protein-like 3 (TCAL3_HUMAN, upregulated), clathrin light chain A (CLCA_HUMAN, upregulated, required for mitotic spindle functioning [63]), centrin-2 (CETN2_HUMAN, upregulated, involved in mRNA export [64]), ribosome biogenesis protein SPATA5L1 (SPA5L_HUMAN, downregulated, an ATP-dependent chaperone, [65]), and telomere-associated protein RIF1 (RIF1_HUMAN, downregulated, key regulator of DNA double-strand break repair [66]). Other proteins found to be dysregulated only upon siRNA treatment against MdmX (Table S3) included transcriptional enhancer factor TEF-3 (TEAD4_HUMAN, downregulated), RNA-binding motif, single-stranded-interacting protein 2 (RBMS2_HUMAN, upregulated), SH3 domain-binding glutamic acid-rich-like protein (SH3L1_HUMAN, upregulated), transcription elongation factor A protein-like 3 (TCAL3_HUMAN, upregulated), and laminin subunit gamma-1 (LAMC1_HUMAN, upregulated).

### 3.2. Gene Ontology and Pathway Enrichment Analysis

**Figure 4:**
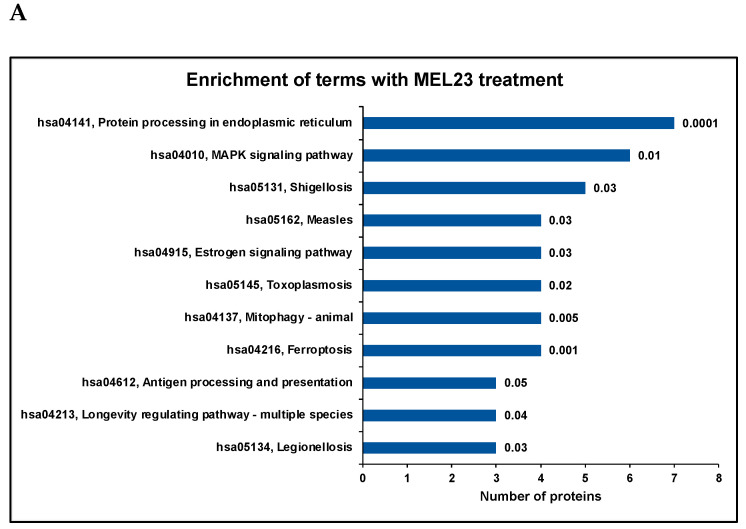

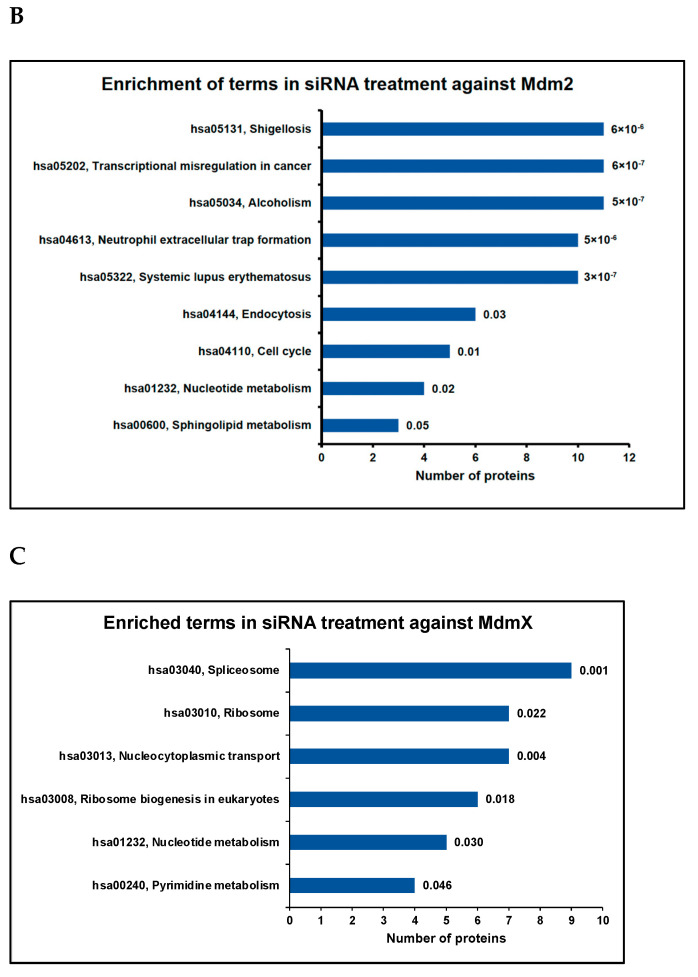
Significantly enriched terms identified in each treatment group are represented by analysis with DAVID using the KEGG database (Fisher exact test). For differential abundance, proteins identified with ≥2 peptides, ≥1.5 or ≤0.667 fold-change, and a post hoc *p*-value < 0.05 are included in the figure. Annotation of each bar has the KEGG term number followed by the KEGG term. Each bar represents the number of proteins associated with that term, and the numbers on the bars represent the *p*-values. In the drug treatment group, proteins dysregulated in the MEL23-treated cells (**A**) compared to DMSO-treated or untreated cells were considered. In the siRNA treatment group, proteins dysregulated in either of the two siRNA-treated cells for each protein (Mdm2 (**B**) and MdmX (**C**)) compared to control siRNA-treated cells are included in the figure. See also Table S4, which provides complementary additional detail such as *p*-values and a listing of each protein’s UniProt ID for each KEGG term represented in this figure.

Gene ontology analysis indicated that protein folding (example proteins include HSP72_HUMAN, DNJB1_HUMAN, and HS71A_HUMAN) and ubiquitin ligase (such as MLP3B_HUMAN, TRAF1_HUMAN, and HS71B_HUMAN) terms were enriched in the MEL23-treated group. The cellular response to stress (with dysregulated proteins such as NUCKS_HUMAN, TRAF1_HUMAN, and RAD51_HUMAN) and protein folding (including proteins HS71A_HUMAN, HS71B_HUMAN, HSP72_HUMAN, DNJA1_HUMAN, and DNJB1_HUMAN) were the enriched biological processes (Figure S1A). Ferroptosis, protein processing in the endoplasmic reticulum, MAPK signaling, and mitophagy were significantly (*p* < 0.05) enriched pathways identified for the differentially abundant proteins in the MEL23-treated cells (Figure 4, Table S4).

In siRNA against Mdm2-treated cells (siMdm2), terms related to pyrophosphate activity (with proteins such as ACSF2_HUMAN, DUT_HUMAN, and ARFG3_HUMAN), chromatin binding (including proteins STAG2_HUMAN, SUZ12_HUMAN, and H1X_HUMAN), cyclin binding (CCNB1_HUMAN, CDK6_HUMAN), etc., were enriched. Cellular component organization (example proteins include SKAP_HUMAN, UBE2C_HUMAN, ARPC5_HUMAN, and TACC3_HUMAN), the cell cycle (with dysregulated proteins including CCNB1_HUMAN, STAG2_HUMAN, PCM1_HUMAN, and CDK6_HUMAN), and the lipid metabolic process (including proteins NSMA3_HUMAN, ACSF2_HUMAN, SPTC2_HUMAN, and HMCS1_HUMAN) were among the biological processes found to be enriched for the differentially abundant proteins (Figure S1B). The cell cycle and endocytosis were two of the significantly enriched (*p* < 0.05) pathways in the siMdm2 dataset (Figure 4, Table S4).

In the siMdmX-treated group, terms related to nucleic acid binding (including proteins such as NOP53_HUMAN, RBM19_HUMAN, SUGP2_HUMAN, CHD4_HUMAN, SRSF5_HUMAN, and LARP4_HUMAN) were enriched. Biological processes enriched included the nucleic acid metabolic process (including proteins such as DDX10_HUMAN, PRP8_HUMAN, POP1_HUMAN, and RNPS1_HUMAN), cellular component organization (example proteins include UBE2C_HUMAN, CLCA_HUMAN, and CCNB1_HUMAN), RNA processing (example proteins include RL7L_HUMAN, RU17_HUMAN, and PESC_HUMAN), and protein transport (including proteins such as IPO11_HUMAN, NU188_HUMAN, and NU107_HUMAN) (Figure S1C). Significantly enriched pathways (*p* < 0.05) in siMdmx were spliceosome, nucleocytoplasmic transport, ribosome, ribosome biogenesis, nucleotide metabolism, and pyrimidine metabolism (Figure 4, Table S4).

### 3.3. Differential Protein Abundance and Discovery of a New Mdm2 Binding Partner

**Figure 5:**
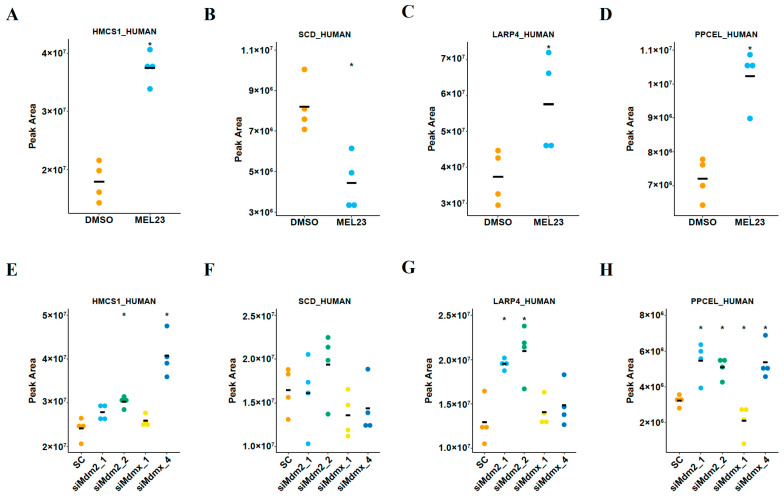
Dot plots of protein quantities as measured by mass spectrometry-based proteomics. Each dot represents one biological replicate. Different colored dots represent the different experimental groups. These proteins were differentially abundant with a fold-change +/−1.5 and post hoc *p*-value < 0.05. Only proteins that meet these criteria for differential abundance and were in common among the treatments indicated were included in the figure. These were SCD_HUMAN (stearoyl-CoA desaturase), HMCS1_HUMAN (hydroxymethylglutaryl-CoA synthase (gene name = HMGCS1), PPCEL_HUMAN (prolyl endopeptidase-like), and LARP4_HUMAN (La-related protein 4). The horizontal black bars represent the mean abundance values (* *p* < 0.05). (**A**–**D**) Protein abundance values in DMSO- and MEL23-treated cells. (**E**–**H**) Protein abundance values in control siRNA-treated cells (SC), Mdm2 siRNA-treated cells (siMdm2_1 and siMdm2_2), and MdmX siRNA-treated cells (siMdmX_1 and siMdmX_4).

Among all significantly differentially abundant proteins, four protein groups were observed in common among the three treatments: stearoyl-CoA desaturase (SCD_HUMAN, O00767), hydroxymethylglutaryl-CoA synthase (HMCS1_HUMAN, Q01581), prolyl endopeptidase-like (PPCEL_HUMAN, Q4J6C6, Q4J6C6-3, Q4J6C6-4), and La-related protein 4 (LARP4_HUMAN, Q71RC2, Q71RC2-4) (Figure 5).

Among those four possible targets, HMCS1_HUMAN (HMGCS1) stood out as an interesting protein since it showed the same trend of modulation in all four siRNA conditions as well as MEL23 treatment, an increase. We then chose this protein to validate the proteomics results by immunoblotting.

Data from mass spectrometry and immunoblotting confirm that HMGCS is upregulated upon inhibition of the activity of the Mdm2-MdmX complex either by pharmacological inhibition or gene silencing (Figure 2A–C). The fact that all three strategies led to a significant increase in HMCS1_HUMAN protein levels points to this protein as a direct target of the Mdm2-MdmX complex. To understand better if that was a possibility, we analyzed if HMCS1_HUMAN physically interacts with the complex in cells, and indeed we found that HMCS1_HUMAN is a binding partner of Mdm2, as seen by co-immunoprecipitation of endogenous protein (Figure 2D). To understand if HMCS1_HUMAN levels increased only at the protein level or if its gene expression was affected as well, we measured the mRNA levels of the HMGCS1 gene. We show that while siMdm2 leads to increased protein levels of HMCS1_HUMAN, gene expression is not significantly changed (Figure 2E). These data describe a new binding partner of Mdm2 and show that the Mdm2-MdmX complex regulates HMCS1_HUMAN at the protein, and not at the RNA level, possibly through ubiquitination of HMCS1_HUMAN, but more experiments are needed to further confirm this hypothesis.

### 3.4. Proteoform Listing

In Tables S1–S3, we indicate instances where distinct proteoforms of a given protein are identified and quantified, using “TRUE” in a column entitled “Distinct Proteoforms Detected”. These proteins map to the same UniProtKB entry name but are classified into different protein groups in the quantitative output from PEAKS.

One interesting example of proteoform-associated differential abundance can be seen in Table S1 for the MEL23 vs. DMSO control. For that comparison, core-binding factor subunit beta (Q13951) is 1.6-fold higher in MEL23-treated cells than in the DMSO control, but is only 1.1-fold increased in abundance for the proteoform listed in UniProt as isoform 2 (Q13951-2) (Figure S6).

For the null comparison of identical HeLa digests at a 250 ng on-column load, no false differential abundance was detected for any proteins that were recorded at +/−1.5-fold with *p* < 0.01. This test of the integrity of the workflow supports the validity of the analysis of H1299 cells (Table S5).

## 4. Discussion

Given its advantage of reproducibility and increased coverage, DIA is becoming the method of choice [16,17,18,19]. To address the challenges in data analysis, newer and improved data analysis tools are being introduced. We used a high-resolution Orbitrap mass spectrometer in the DIA mode and analyzed the data with PEAKS Studio.

With this analytical pipeline, we evaluated how changes in the Mdm2-MdmX complex effect H1299 cells’ proteomics profile. Considering that Mdm2 and MdmX can work together as a complex or individually [11], we used two different approaches to modify the activity of the Mdm2-MdmX complex: a molecular approach using siRNAs to knockdown each component of the complex separately, and a functional approach using the inhibitor MEL23, where both proteins are present but do not form an active E3 ligase complex. We identified some differentially abundant proteins exclusive to each treatment type, suggesting that certain processes could be linked to E3 ligase activity of the Mdm2-MdmX complex while some may be independent of it and instead associated with E3-ligase-independent functions.

Differentially abundant proteins associated with protein folding and processing were found upon modification of Mdm2-MdmX E3 ligase activity. These include heat shock proteins, co-chaperones, and proteins associated with endoplasmic reticulum-associated degradation of proteins. We identified molecular chaperones DnaJ homolog subfamily A member 1 (DNJA1_HUMAN), DnaJ homolog subfamily B member 1 (DNJB1_HUMAN), and heat shock protein 70 (HS71A_HUMAN, HS71B_HUMAN) as upregulated. These proteins have been shown to stabilize misfolded or mutated p53 [67,68,69,70]. Further studies will be required to elucidate their relationship with Mdm2 or MdmX. As has been reported earlier, Mdm2 also facilitates mitophagy by enhancing parkin activity [71]. Though we did not identify parkin in our dataset, the mitophagy pathway was found to be enriched in the differentially abundant proteins.

Abundance levels of proteins involved in endocytosis and the cell cycle were dysregulated upon downregulation of Mdm2 abundance. Also, proteins associated with the cell cycle and DNA damage repair (CD123_HUMAN, SDE2_HUMAN, RAD51_HUMAN, BUB1B_HUMAN, CHK1_HUMAN) were found to be upregulated upon MEL23 treatment. This suggests a cell cycle regulatory function of the Mdm2-MdmX complex or Mdm2 alone, which has also been reported [72]. Chromatin binding, cyclin binding, and rRNA binding were some of the enriched molecular function categories for dysregulated proteins upon downregulation of Mdm2 levels. The chromatin-binding and -modifying function of Mdm2 has been reported to regulate not only DNA damage but also amino acid metabolism and stemness in pluripotent cells [73,74,75,76]. These results, along with previously reported roles of Mdm2 in DNA damage repair and regulation of genome stability, also substantiate the cell cycle regulatory role of Mdm2 in the DNA damage response or genome stability as an important aspect of cell cycle regulation [77,78,79].

Separate studies have shown that Mdm2-mediated ubiquitination of β-arrestin-2 or PKCβII can drive clathrin-dependent endocytosis [80,81]. MdmX downregulation affected the abundance of many spliceosomal proteins, ribosomal proteins, and proteins involved in nucleocytoplasmic transport, ribosome biogenesis, and pyrimidine metabolism, with most of them showing downregulation. Many ribosomal proteins, such as L23, L11, L37, and L42, have been shown to interact with Mdm2, but this binding of ribosomal protein with Mdm2 has been reported as a mechanism to regulate the p53-Mdm2 pathway [82,83,84,85].

It is noteworthy that the downregulation of Mdm2 and MdmX resulted in the alteration of different proteins. However, this can be explained by the fact that Mdm2 and MdmX have been known to play several independent roles. Four proteins were found to be differentially abundant in common in the three datasets. Of these four proteins, stearoyl-CoA desaturase (SCD_HUMAN, downregulated upon MEL23 treatment while being downregulated upon siRNA against Mdm2 and MdmX treatment) and hydroxymethylglutaryl-CoA synthase (HMCS1_HUMAN, upregulated in all the three treatment groups) found to be involved in peroxisome proliferator-activated receptor (PPAR) signaling upon gene ontology analysis. Stearoyl-CoA desaturase is an integral membrane protein present in the endoplasmic reticulum and acts as an enzyme, converting saturated fatty acids to monounsaturated fatty acids [53]. Several studies have previously reported the role of SCD1 in ferroptosis [52,86,87]. Hydroxymethylglutaryl-CoA synthase is an important enzyme in the mevalonate pathway. The mevalonate pathway has been reported as a crucial signaling network in ferroptosis induction by providing isopentenyl pyrophosphate [58,88]. Thus, two out of four proteins found to be regulated by the inhibition of the Mdm2-MdmX complex are somehow associated with ferroptosis. We also found that proteins involved in the ferroptosis pathway were dysregulated upon MEL23 treatment. These include microtubule-associated proteins 1A/1B light chain 3B (MP3B2_HUMAN, MLP3B_HUMAN), long-chain fatty acid-CoA ligase 4 (ACSL4_HUMAN), and phospholipid hydroperoxide glutathione peroxidase (GPX4_HUMAN), the key regulatory protein of ferroptosis. Indeed, a recent work reports that Mdm2 and MdmX promote ferroptosis via PPARα-mediated lipid remodeling [89].

### 4.1. Limitations of This Study

Our study has limitations. These include the ever-present limitations in sensitivity of mass spectrometry equipment. We at least partly ameliorated that limitation by using the more sensitive DIA method as well as PEAKS software, which has a robust match-between-runs capability, serving to further enhance sensitivity for low-abundance proteins. Other limitations in shotgun proteomics studies include proteome complexity based on the levels of abundance of particular protein species, such as amino acid sequence variants or posttranslational modifications, resulting in proteoforms that might have different abundance patterns.

An additional point to address is the assessment of protein levels by Western blotting. Beyond protein quantification (method #1), conducted after preparing cell lysates, we employed β-actin as a loading control (method #2) to ensure equal sample loading across lanes. While new, sophisticated imaging systems that assess the total protein content have been proposed as alternatives to traditional loading controls, they also have limitations. Despite the existence of various methods to evaluate protein amounts, the use of loading controls remains a widely accepted standard for qualitative protein analysis. One limitation of this approach is whether β-actin can be consistently relied upon as invariant across all experimental conditions. However, the extensive literature supporting the use of β-actin as a loading control in diverse experimental contexts, coupled with the consistent signals observed between lanes loaded with equal amounts of protein (as determined by protein quantification), supports its validity in our study.

Another consideration is the efficiency of protein transfer to the blot membrane, which could potentially lead to misinterpretation. In our case, this limitation is diminished because we compare only the relative protein levels within the same immunoblot membrane. Even if transfer efficiency varied, it would uniformly affect the entire membrane, ensuring proportional loss across all lanes. Consequently, the observed results remain reliable. Thus, by using immunoblotting, even in the face of any limitations, we were able to demonstrate the qualitative changes in protein abundance as intended in this study.

Some bands in Figure S7 might indicate multiple reactivity of the antibodies used (poor antibody selectivity/quality), the nature of the protocol used, and/or the likely presence of proteoforms.

Other limitations include the need for follow-up studies that could validate and expand upon the reported abundance changes. These include targeted LC-MS/MS of relevant peptides and validation of predicted protein–protein interactions using orthogonal methods such as cross-linking or proximity labeling to increase the impact of this work. Such studies, whether conducted by our lab or independent investigators, would corroborate and strengthen our findings, with independent replication serving as the gold standard for validation.

It is known that drugs and small molecules often present off-target effects [90] as well as siRNAs [91]. Important to note is that while we were able to find similarities between the treatments performed in this study, each protocol (drug treatment or siRNA transfection) possesses off-target effects at some degree, which possibly accounts for some of the differences we see in the proteome of those samples. Here, we focused on communalities since all three strategies disrupt the E3-ligase function of the Mdm2-X complex, which was the focus of this work.

Possible off-target or nonspecific effects might contribute to the observed proteome differences between MEL23 treatment and gene knockdown, but this paper is focused on the analysis of p53-independent functions of the Mdm2-MdmX complex using data-independent acquisition-based profiling, and it was mostly a mass-spectrometry-based study. Attempting to uncover possible off-target effects of MEL23 or siRNA on the proteome could be included in follow-up work.

### 4.2. Summary

We have identified pathways and biological processes affected by modification of Mdm2-MdmX activity, which have not been described as related to p53, reinforcing the idea that this complex plays a role in cellular processes in a p53-ndependent manner. Though some of these functions have been reported earlier, we have found new targets and links that could help us better understand those correlations. Additionally, we report pathways that were differentially affected by the regulation of Mdm2 or MdmX alone but not by the inhibition of the E3 ligase complex, which can help us to understand the roles of these two proteins as monomers, independent of the formation of a functional complex.

We also identify a new binding partner of Mdm2, HMCS1_HUMAN, in lung adenocarcinoma cells.

### 4.3. Future Studies

Further studies are required to define and allow us to better understand these complex-dependent and -independent roles, and well as the biological function of this newly described interaction between Mdm2 and HMCS1_HUMAN.

## Data Availability

The MS-based proteomic data have been deposited at the MassIVE repository under identifier MSV000093577.

## Supplementary Materials

The following supporting information can be downloaded at https://www.mdpi.com/article/10.3390/proteomes13020018/s1, Figure S1: Bar graphs representing the number of proteins enriched in each category for molecular functions (orange) and biological processes (blue) for differentially abundant proteins in (A) MEL23-treated cells, (B) siRNA against Mdm2-treated cells, and (C) siRNA against MdmX-treated cells; Figure S2: Evidence supporting the identification of SCD_HUMAN (stearoyl-CoA desaturase) with a coverage map (upper) and fragmentation spectra (lower) for two example peptides; Figure S3: Evidence supporting the identification of HMCS1_HUMAN (hydroxymethylglutaryl-CoA synthase) (gene name = HMGCS1) with a coverage map (upper) and fragmentation spectra (lower) for two example peptides; Figure S4: Evidence supporting the identification of PPCEL_HUMAN (prolyl endopeptidase-like) with a coverage map (upper) and fragmentation spectra (lower) for two example peptides; Figure S5: Evidence supporting the identification of LARP4_HUMAN (La-related protein 4) with a coverage map (upper) and fragmentation spectra (lower) for two example peptides; Figure S6: Proteoforms of core-binding factor subunit beta (PEBB_HUMAN) with MEL23 treatment compared to DMSO control; Figure S7: Image of original immunoblot; Table S1: Proteins differentially regulated upon MEL23 treatment; Table S2: Proteins differentially regulated upon siRNA treatment against Mdm2; Table S3: Proteins differentially regulated upon siRNA treatment against MdmX; Table S4. Significantly enriched terms identified in each treatment group are represented in the table by analysis with DAVID using the KEGG database (Fisher Exact test); Table S5. To test the workflow and guard against spurious false positives, a null trial was performed with a HeLa digest divided in half; Table S6. Densitometry measurements for the immunoblot.
