# Supplementary material for: Analysis of p53-Independent Functions of the Mdm2-MdmX Complex Using Data-Independent Acquisition-Based Profiling
Source: Proteomes. 2025 May 22;13(2):18. doi: 10.3390/proteomes13020018 (PMC12196705; doi:10.3390/proteomes13020018)

Figure S7 – Original unprocessed western blot images corresponding to Figure 2

Analysis of p53-Independent Functions of the Mdm2-MdmX Complex Using Data-Independent Acquisition-Based Profiling

Anu Jain 1,2, Rafaela Muniz de Queiroz 1, Jayanta K. Chakrabarty 1,2, Karl A. T. Makepeace 1,2, Carol Prives 1

and Lewis M. Brown 1,2,*

Figure 2A -MdmX

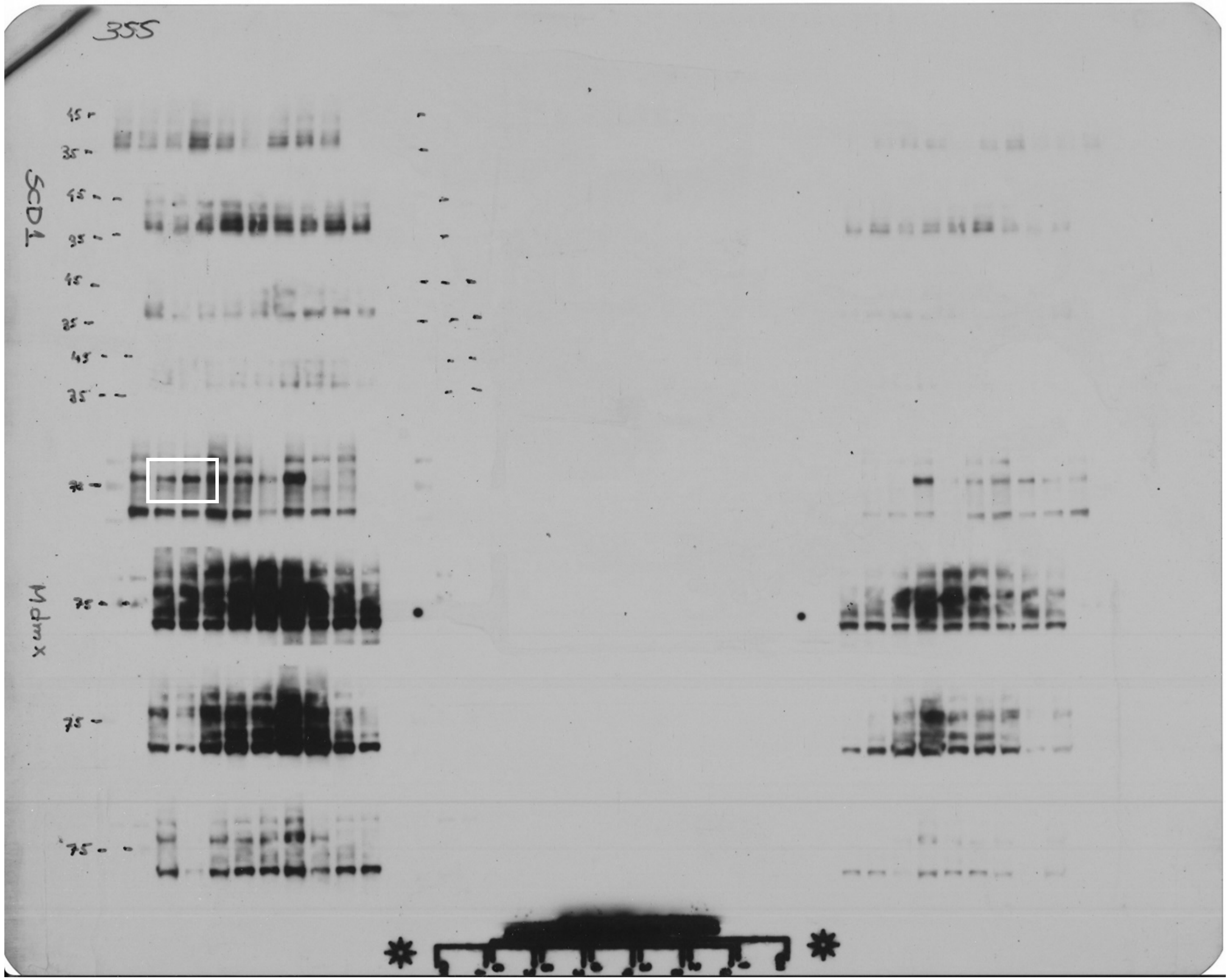

Figure 2A –Mdm2

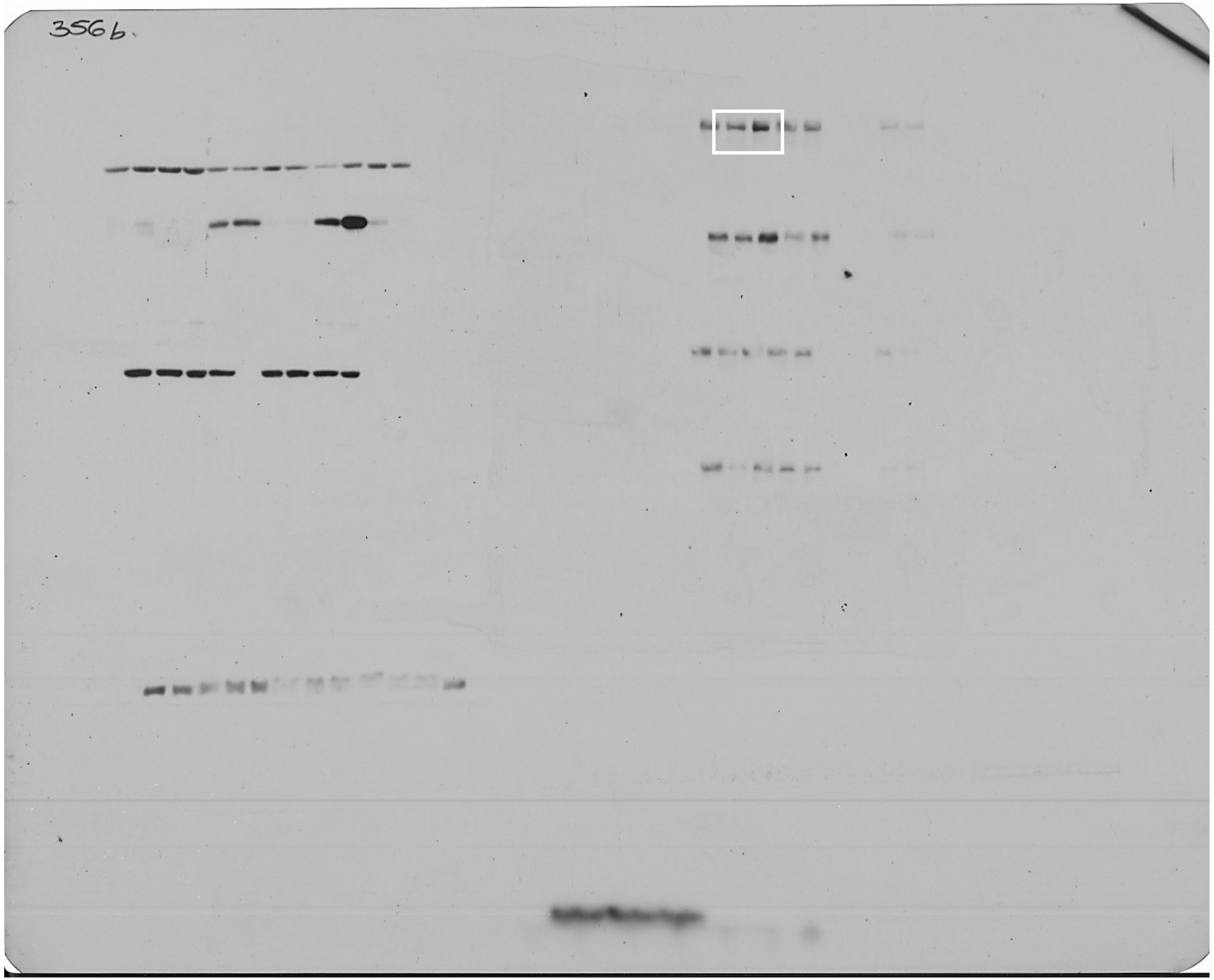

Figure 2A -Actin

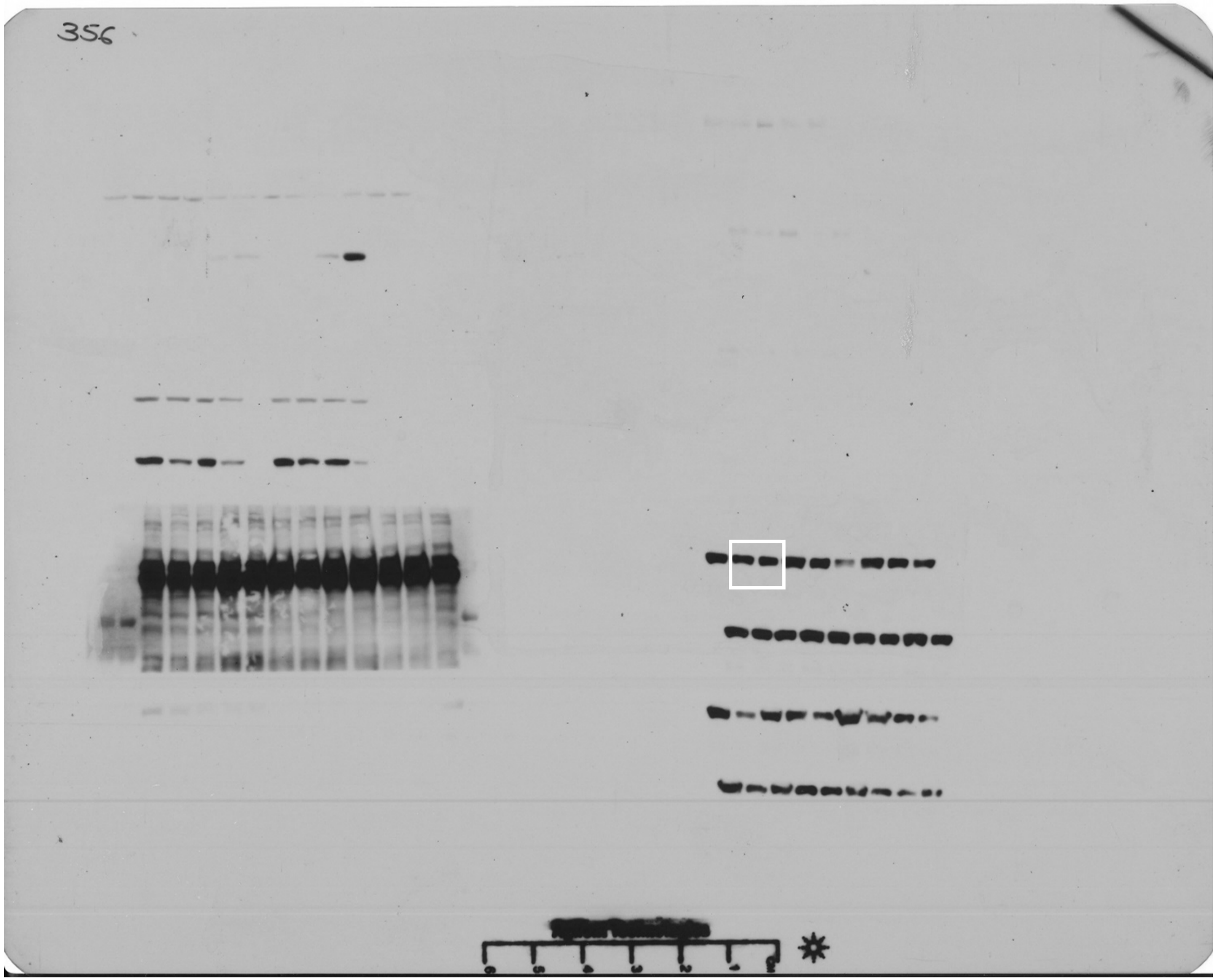

Figure 2B -Mdm2

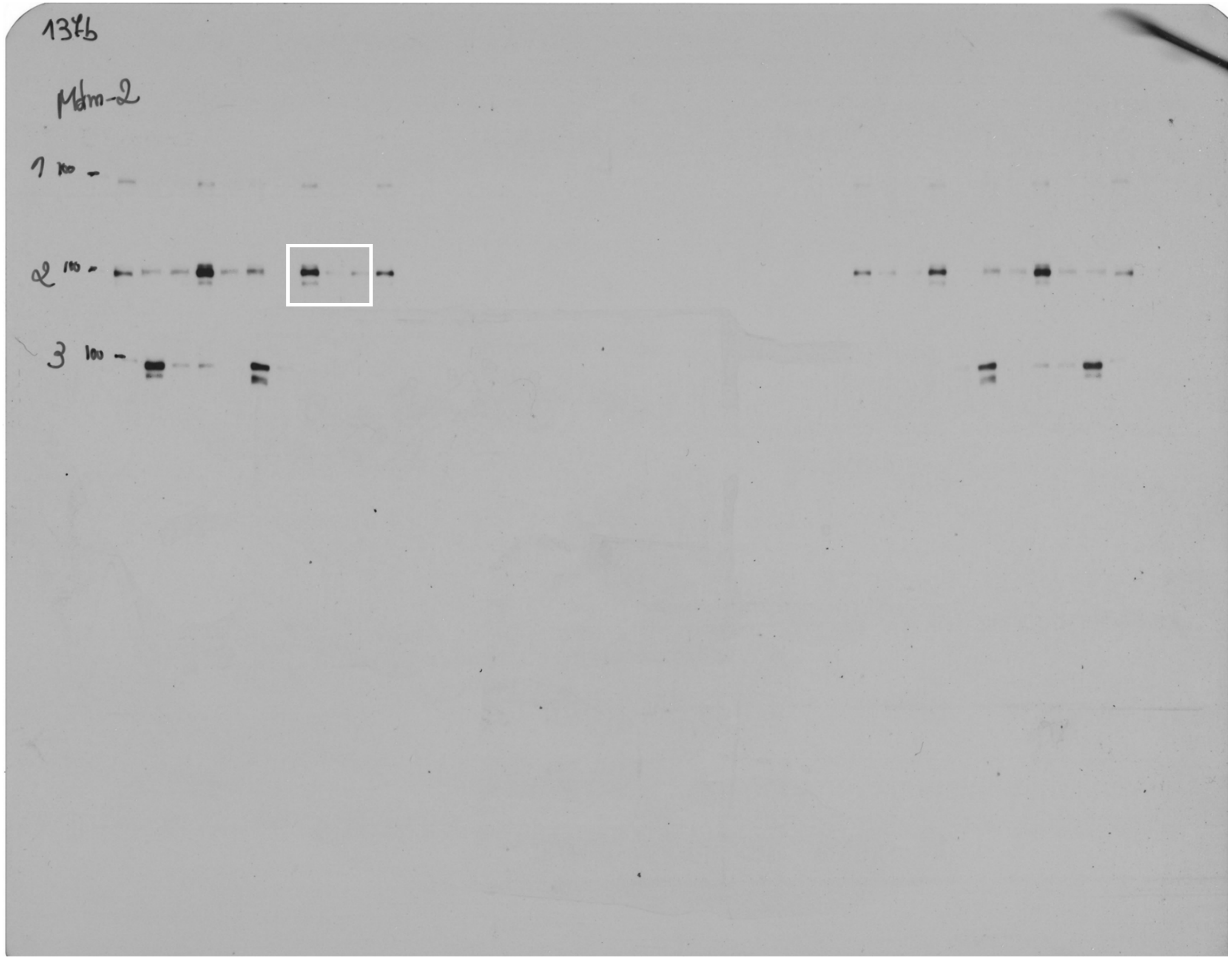

Figure 2B -Actin

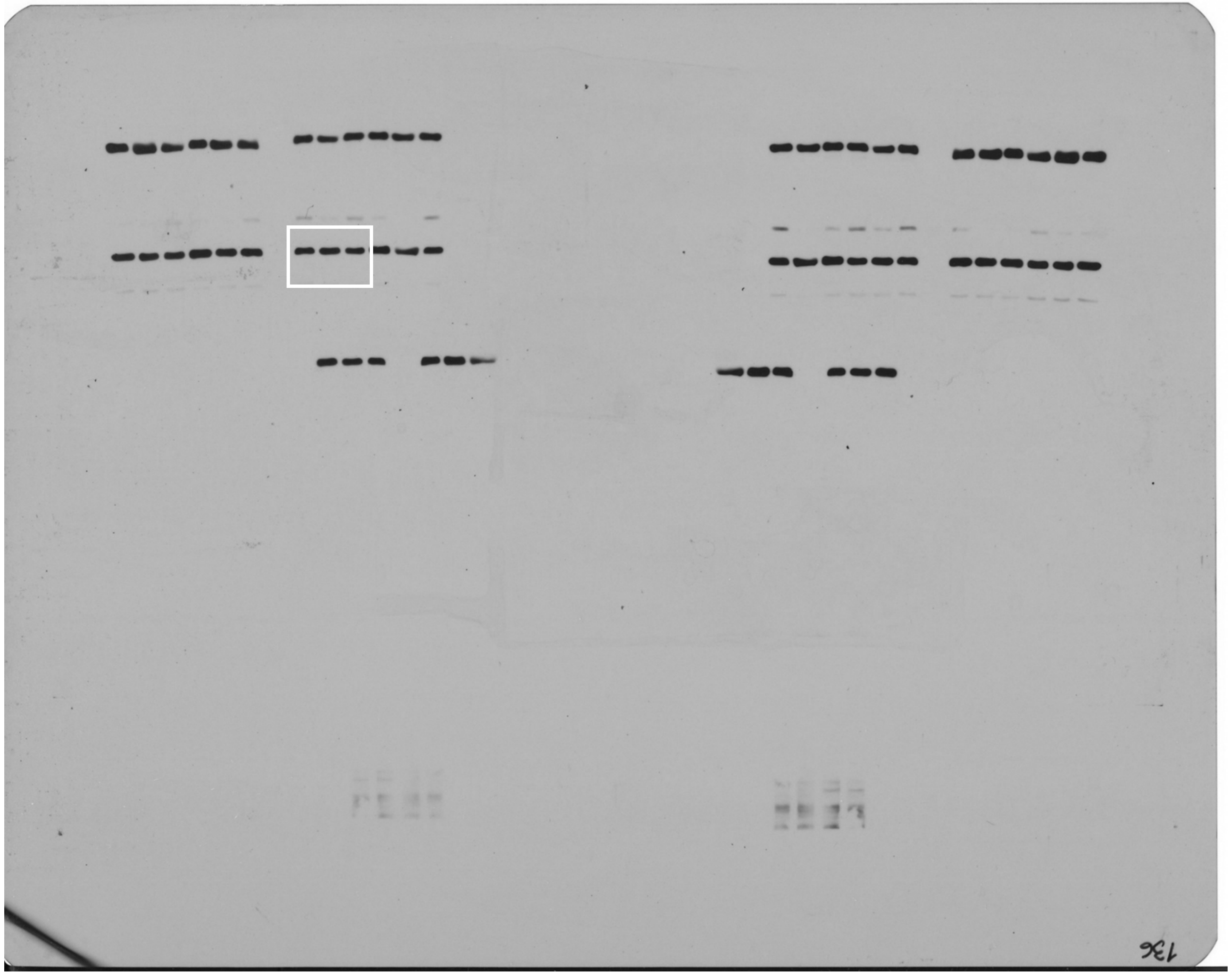

Figure 2C -MdmX

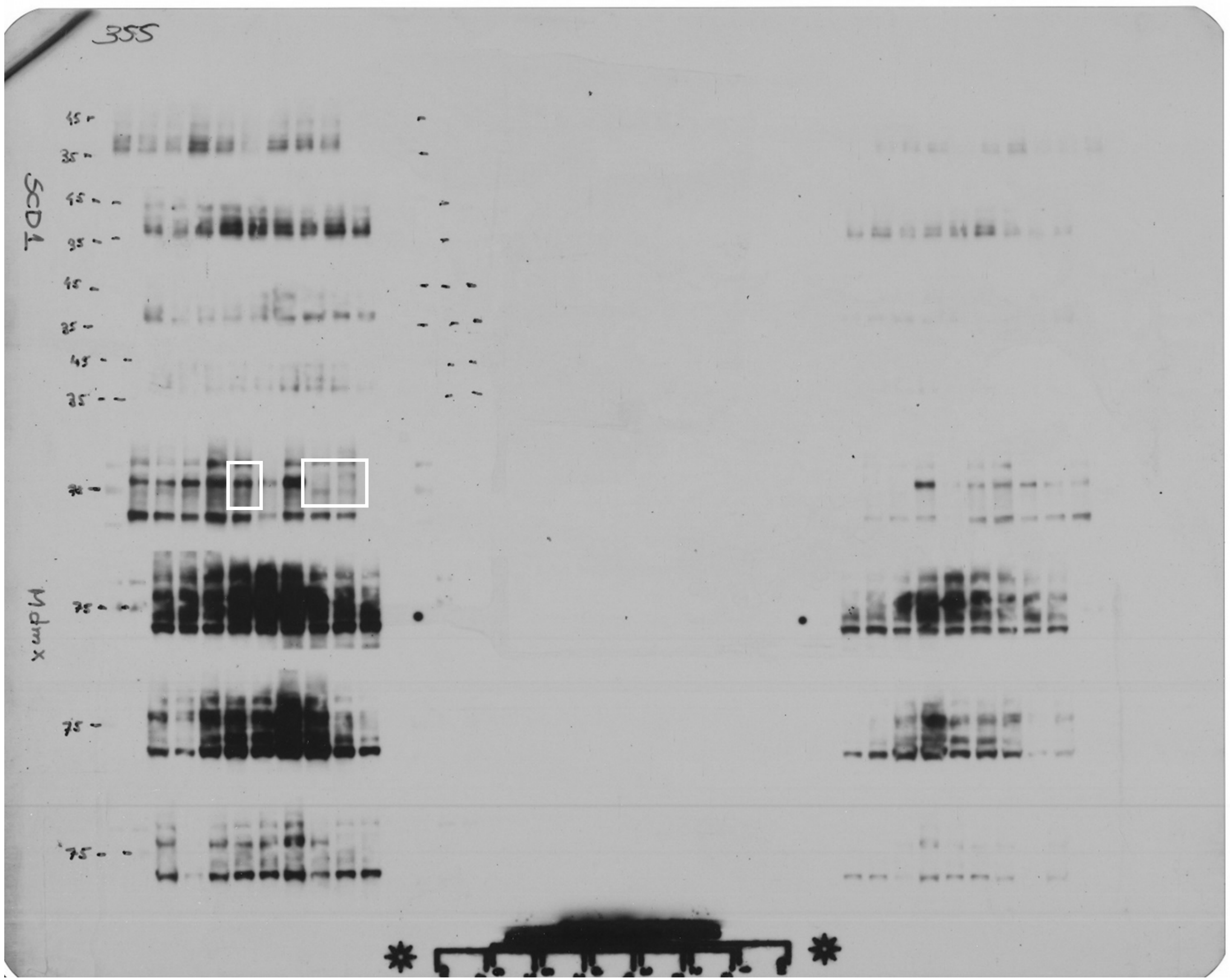

Figure 2C -Actin

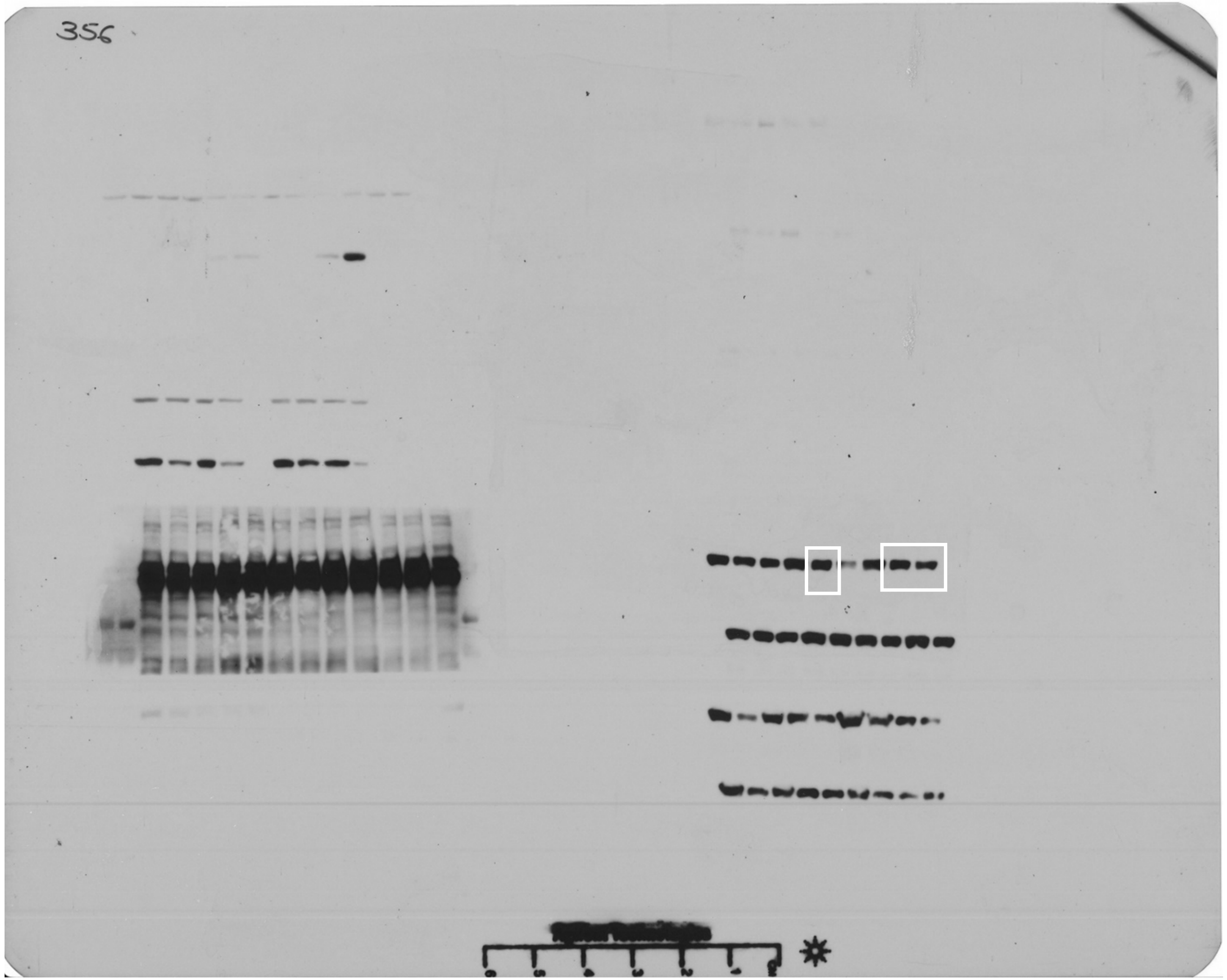

Figure 2D –HMGCS1 (short exposure)

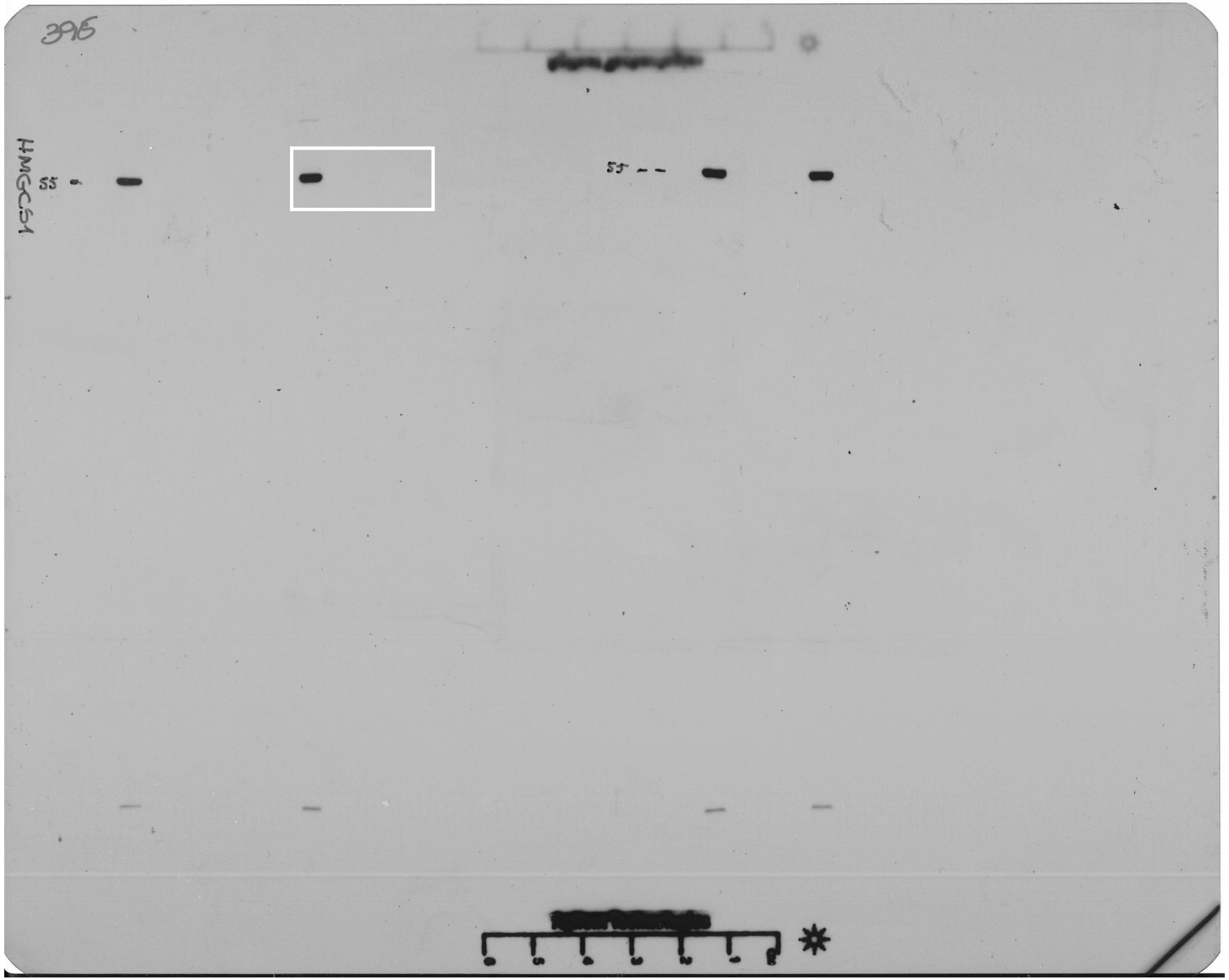

Figure 2D –HMGCS1 (long exposure)

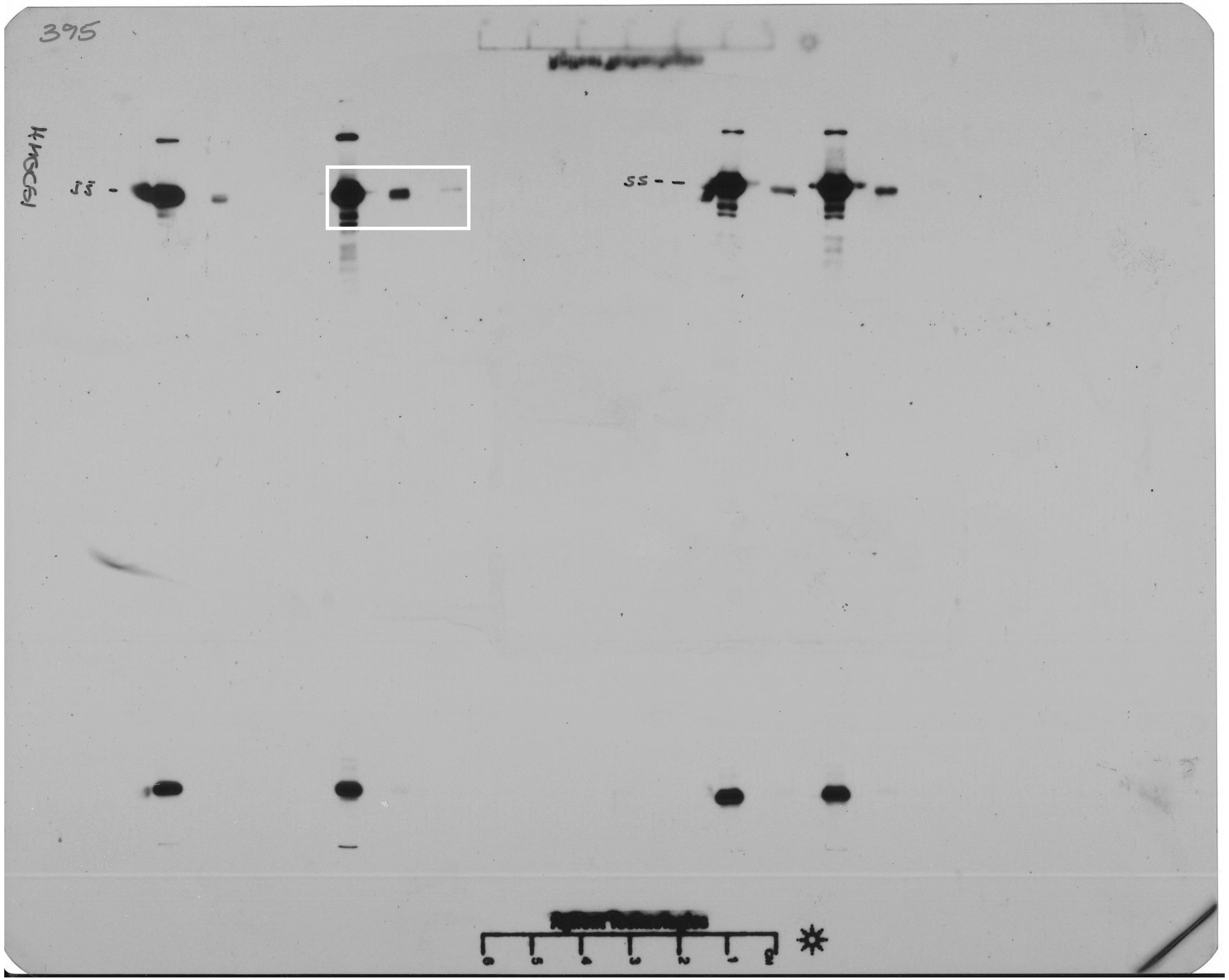

Figure 2D –Mdm2

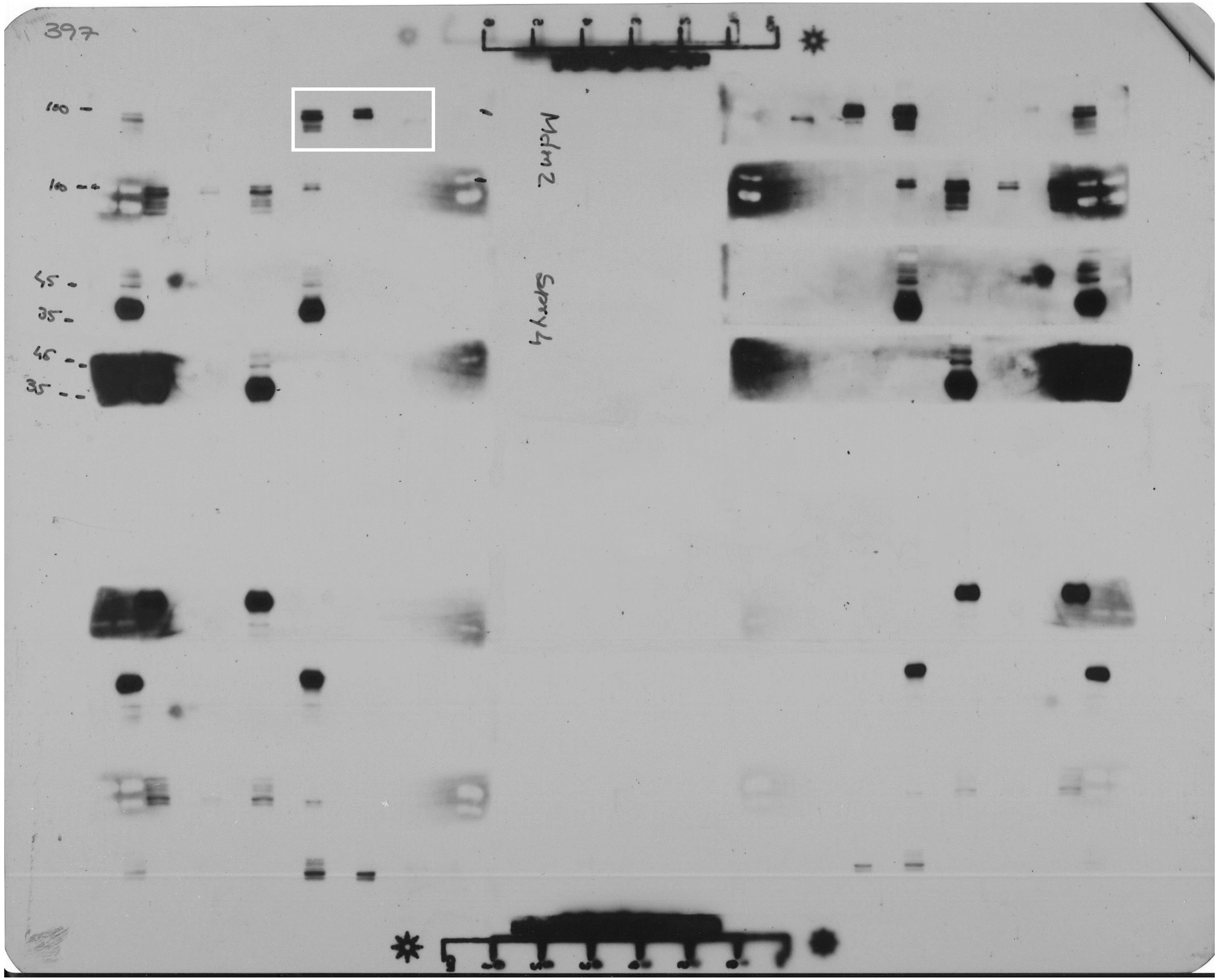

Supplement: Supplementary file 1 [file proteomes-13-00018-s001.zip › Jain_et_al_Fig_S7.pdf]
