# Supplementary material for: Analysis of p53-Independent Functions of the Mdm2-MdmX Complex Using Data-Independent Acquisition-Based Profiling
Source: Proteomes. 2025 May 22;13(2):18. doi: 10.3390/proteomes13020018 (PMC12196705; doi:10.3390/proteomes13020018)
Supplement: Supplementary file 1 [file proteomes-13-00018-s001.zip › supplementary materials Figures S2-S6 and Table S4.pdf]

```
  1  MPAHLLQDDI SSSYTTTTTI TAPPSRVLQN GGDKLETMPL YLEDDIRPDI KDDIYDPTYK DKEGPSPKVE YVWRNIILMS
 81  LLHLGALYGI TLIPTCKFYT WLWGVFYYFV SALGITAGAH RLWSHRSYKA RLPLRLFLII ANTMAFQNDV YEWARDHRAH
161  HKFSETHADP HNSRRGFFFS HVGWLLVRKH PAVKEKGSTL DLSDLEAEKL VMFQRRYYKP GLLMMCFILP TLVPWYFWGE
241  TFQNSVFVAT FLRYAVVLNA TWLVNSAAHL FGYRPYDKNI SPRENILVSL GAVGEGFHNY HHSFPYDYSA SEYRWHINFT
321  TFFIDCMAAL GLAYDRKKVS KAAILARIKR TGDGNYKSG
```

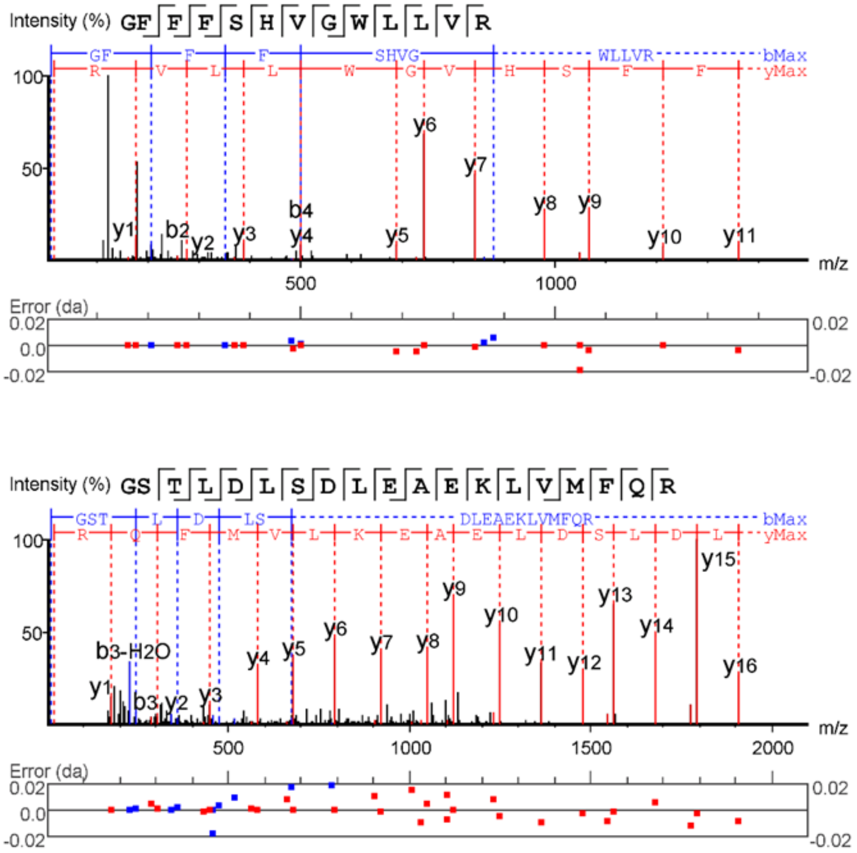

Figure S2: Evidence supporting the identification of SCD_HUMAN (stearoyl-CoA desaturase) with coverage map (upper) and fragmentation spectra (lower) for two example peptides.

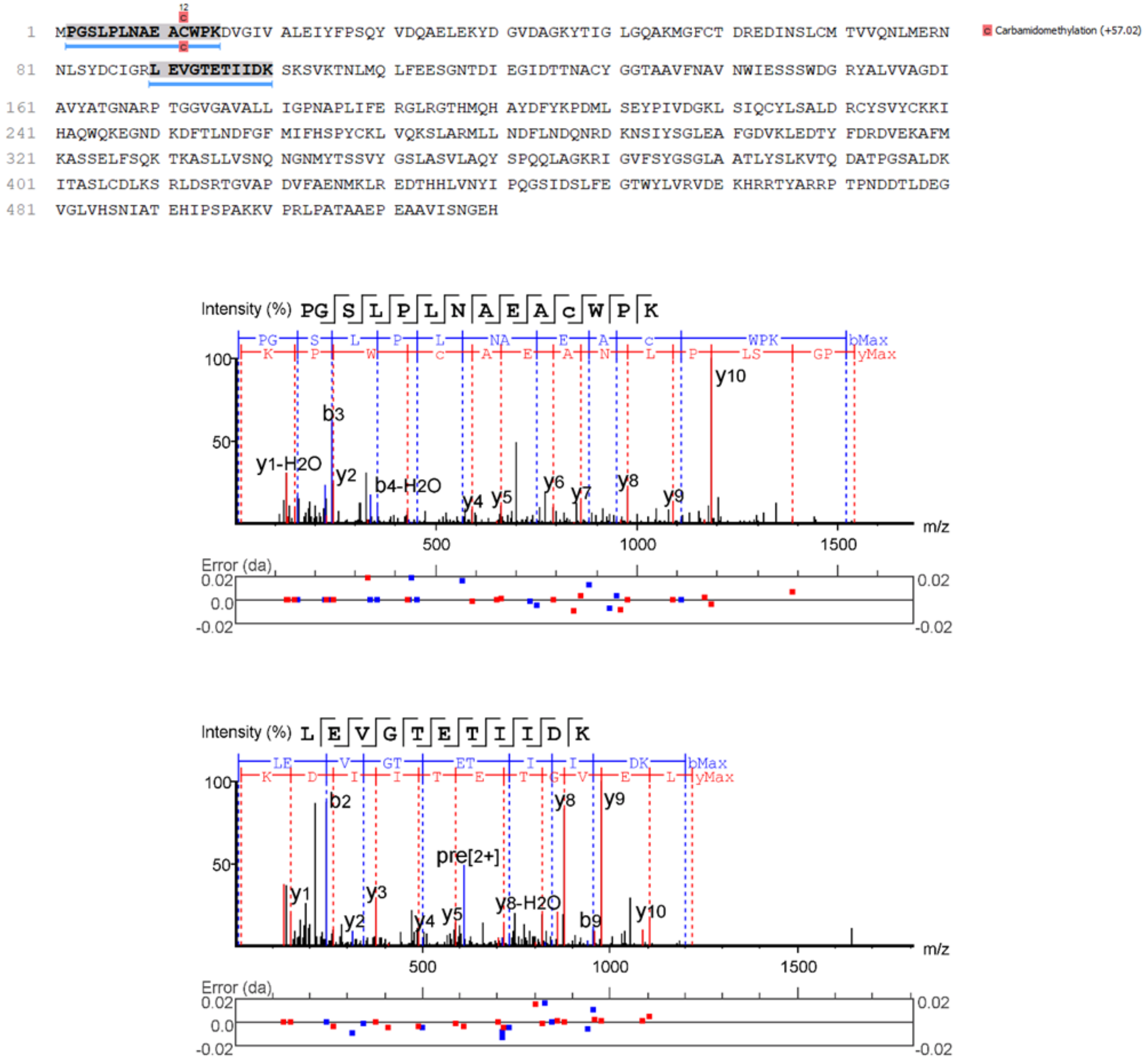

Figure S3: Evidence supporting the identification of HMCS1_HUMAN (hydroxymethylglutaryl-CoA synthase (gene name = HMGCS1) with coverage map (upper) and fragmentation spectra (lower) for two example peptides.

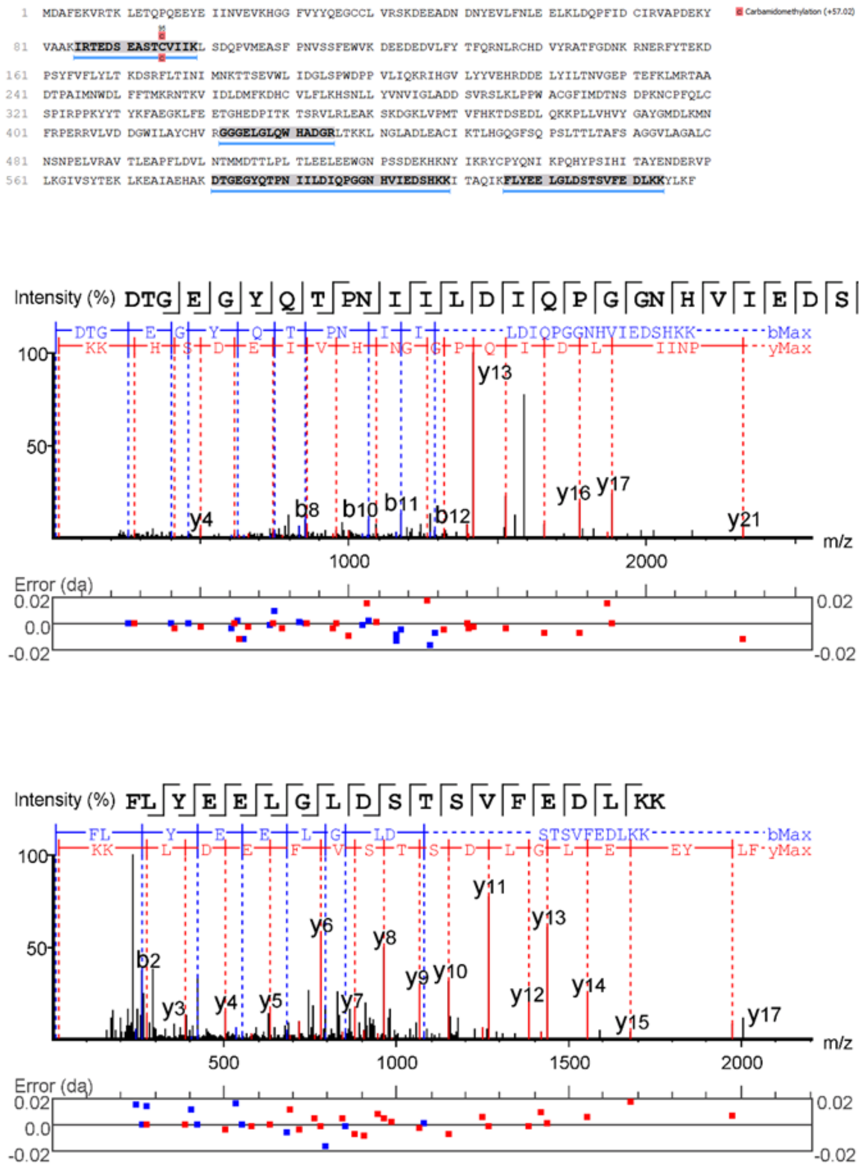

Figure S4: Evidence supporting the identification of PPCEL_HUMAN (prolyl endopeptidase-like) with coverage map (upper) and fragmentation spectra (lower) for two example peptides.

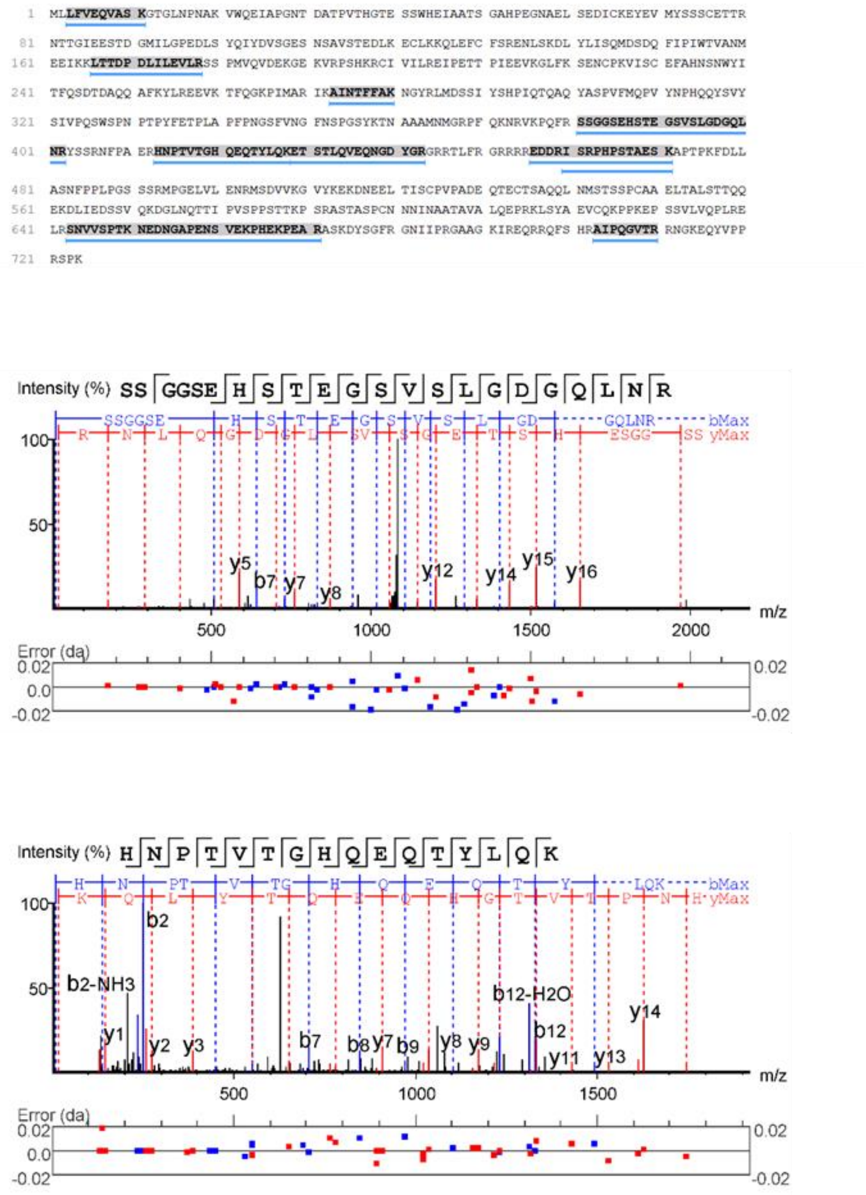

Figure S5: Evidence supporting the identification of LARP4_HUMAN (La-related protein 4) with coverage map (upper) and fragmentation spectra (lower) for two example peptides.

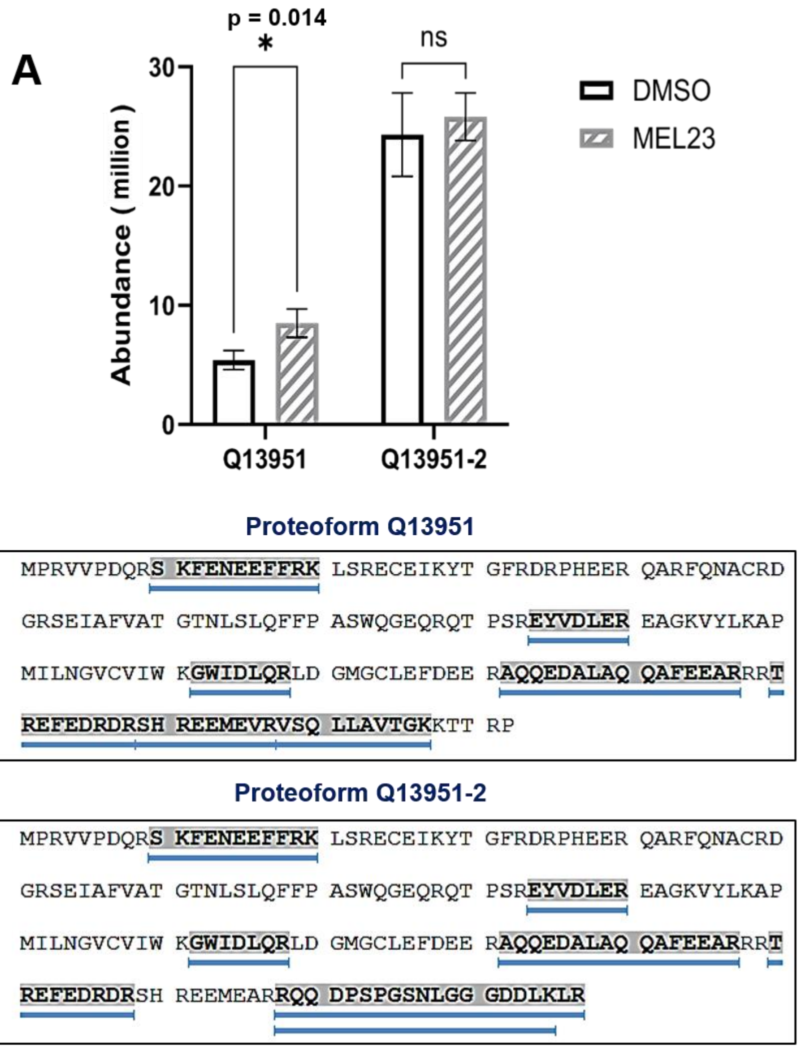

C

Q13951 Peptides	Quality Score	Q13951-2 Peptides	Quality Score
R.SKFENEEFFRK.L	6.9	R.SKFENEEFFRK.L	6.9
R.EYVDLER.E	23.3	R.EYVDLER.E	23.3
K.GWIDLQR.L	23.9	K.GWIDLQR.L	23.9
R.AQQEDALAQQAFEEAR.R	10.5	R.AQQEDALAQQAFEEAR.R	10.5
R.TREFEDRDR.S	15.0	R.TREFEDRDR.S	15.0
R.SHREEMEVR.V	7.3		
R.VSQLLAVTGK.K	21.6		
		R.RQQDPSPGSNLGGGDDLK.L	16.4
		R.RQQDPSPGSNLGGGDDLKLR	6.6

Figure S6: Proteoforms of core-binding factor subunit beta (PEBB_HUMAN) with MEL23 treatment compared to DMSO control. (A) Mass spectrometry quantitative abundance of the canonical proteoform (Q13951) compared to a detected variant proteoform (Q13951-2) (Means ±SD). (B) Amino acid sequence of each proteoform as determined by PEAKS software. (C) PEAKS identification quality score for each peptide detected in each proteoform.

Table S4. Significantly enriched terms identified in each treatment group are represented in the table by analysis with DAVID using the KEGG database (Fisher Exact test). For differential expression, proteins identified with ≥2 peptides, ≥1.5 and ≤0.667 fold-change with post-hoc p-value <0.05 were listed in the table. In the drug treatment group, proteins dysregulated in the MEL23 treated cells compared to DMSO treated or untreated cells were considered. In the siRNA treatment group, proteins dysregulated in either of the two siRNA-treated cells for each protein (Mdm2 and Mdmx) compared to control siRNA-treated cells were listed in the table. Proteins dysregulated the cells treated with the different siRNA are color-coded as described below.

Treatment	Term	Name	Count	%	p-value	UniProt ID
MEL23	hsa04141	Protein processing in endoplasmic reticulum	7	9	1E-04	UBXN4_HUMAN, HS71A_HUMAN, UB2D3_HUMAN, HSP72_HUMAN, DNJB1_HUMAN, DNJA1_HUMAN, HS71B_HUMAN
	hsa04216	Ferroptosis	4	5	1E-03	MP3B2_HUMAN, MLP3B_HUMAN, GPX4_HUMAN, ACSL4_HUMAN
	hsa04137	Mitophagy - animal	4	5	5E-03	MP3B2_HUMAN, MLP3B_HUMAN, JUN_HUMAN, CACO2_HUMAN
	hsa04010	MAPK signaling pathway	6	8	0.01	STK3_HUMAN, HS71A_HUMAN, TGFB1_HUMAN, HSP72_HUMAN, JUN_HUMAN, HS71B_HUMAN
	hsa05145	Toxoplasmosis	4	5	0.02	HS71A_HUMAN, TGFB1_HUMAN, HSP72_HUMAN, HS71B_HUMAN
	hsa04915	Estrogen signaling pathway	4	5	0.03	HS71A_HUMAN, HSP72_HUMAN, JUN_HUMAN, HS71B_HUMAN
	hsa05162	Measles	4	5	0.03	HS71A_HUMAN, HSP72_HUMAN, JUN_HUMAN, HS71B_HUMAN
	hsa05131	Shigellosis	5	6	0.03	MP3B2_HUMAN, MLP3B_HUMAN, UB2D3_HUMAN, JUN_HUMAN, CACO2_HUMAN
	hsa05134	Legionellosis	3	4	0.03	HS71A_HUMAN, HSP72_HUMAN, HS71B_HUMAN
	hsa04213	Longevity regulating pathway - multiple species	3	4	0.04	HS71A_HUMAN, HSP72_HUMAN, HS71B_HUMAN
	hsa04612	Antigen processing and presentation	3	4	0.05	HS71A_HUMAN, HSP72_HUMAN, HS71B_HUMAN
siRNA against Mdm2	hsa05322	Systemic lupus erythematosus	10	9.4	3E-07	H31_HUMAN
	hsa05034	Alcoholism	11	10.4	5E-07	H31_HUMAN, SHC1_HUMAN
	hsa05202	Transcriptional misregulation in cancer	11	10.4	6E-07	H31_HUMAN, TRAF1_HUMAN
	hsa04613	Neutrophil extracellular trap formation	10	9.4	5E-06	H31_HUMAN
	hsa05131	Shigellosis	11	10.4	6E-06	ARPC5_HUMAN, H31_HUMAN
	hsa04110	Cell cycle	5	4.7	0.01	SMAD3_HUMAN, CDK6_HUMAN, CDC20_HUMAN, STAG2_HUMAN, CCNB1_HUMAN
	hsa01232	Nucleotide metabolism	4	3.8	0.02	PNPH_HUMAN, DUT_HUMAN, PURA2_HUMAN, 5NTC_HUMAN
	hsa04144	Endocytosis	6	5.7	0.03	SMAD3_HUMAN, ARPC5_HUMAN, ARFG3_HUMAN, SH3K1_HUMAN, RAB5C_HUMAN, STAM2_HUMAN
	hsa00600	Sphingolipid metabolism	3	2.8	0.05	NSMA3_HUMAN, CERS2_HUMAN, SPTC2_HUMAN
siRNA against MdmX	hsa03040	Spliceosome	9	3.6	0.001	SRSF5_HUMAN, DHX16_HUMAN, HNRPU_HUMAN, THOC2_HUMAN, SNUT1_HUMAN, LSM3_HUMAN, PRP8_HUMAN, RU17_HUMAN, RBMX_HUMAN
	hsa03013	Nucleocytoplasmic transport	7	2.8	0.004	RNPS1_HUMAN, NUP58_HUMAN, NU153_HUMAN, IPO11_HUMAN,

						THOC2_HUMAN, NU107_HUMAN, NU188_HUMAN
	**hsa03008**	**Ribosome biogenesis in eukaryotes**	**6**	**2.4**	**0.018**	**UT14A_HUMAN, POP1_HUMAN, NAT10_HUMAN, RBM28_HUMAN, UTP18_HUMAN, WDR36_HUMAN**
	**hsa03010**	**Ribosome**	**7**	**2.8**	**0.022**	**RL37A_HUMAN, RM09_HUMAN, RL23_HUMAN, RL14_HUMAN, RL37_HUMAN, RS13_HUMAN, RL26L_HUMAN**
	**hsa01232**	**Nucleotide metabolism**	**5**	**2.0**	**0.030**	**TYSY_HUMAN, KITH_HUMAN, RIR2_HUMAN, 5NTC_HUMAN, KAD3_HUMAN**
	**hsa00240**	**Pyrimidine metabolism**	**4**	**1.6**	**0.046**	**TYSY_HUMAN, KITH_HUMAN, RIR2_HUMAN, 5NTC_HUMAN**

For Mdm2

Blue highlighted: dysregulated in siRNA1

Grey highlighted: dysregulated in siRNA2

No overlap in both the siRNAs

For Mdmx

Blue highlighted: dysregulated in siRNA4

Grey highlighted: dysregulated in siRNA1

Yellow highlighted: dysregulated in both
