# Supplementary figures and images for: Analysis of p53-Independent Functions of the Mdm2-MdmX Complex Using Data-Independent Acquisition-Based Profiling
Source: Proteomes. 2025 May 22;13(2):18. doi: 10.3390/proteomes13020018 (PMC12196705; doi:10.3390/proteomes13020018)

## Supplementary figure 1

A

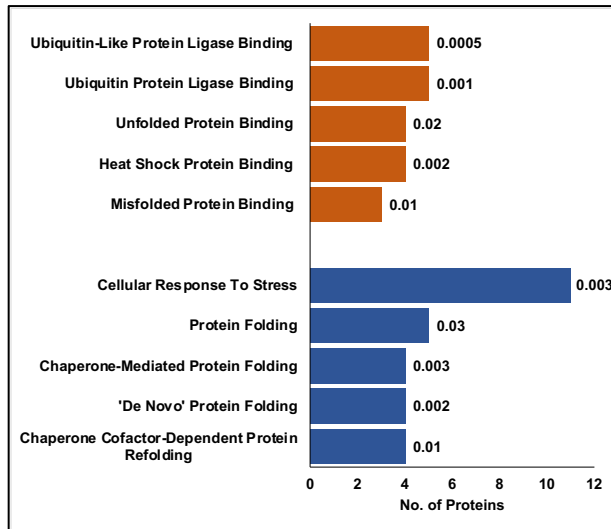

B

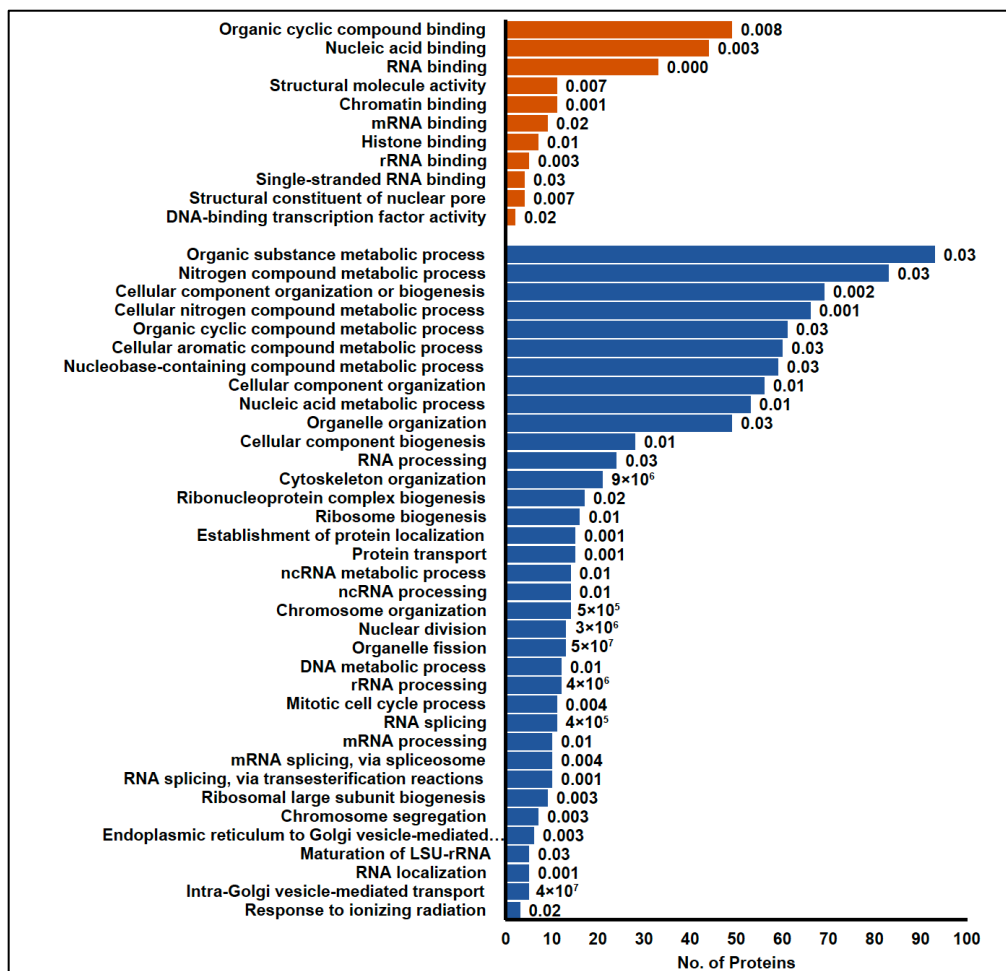

C

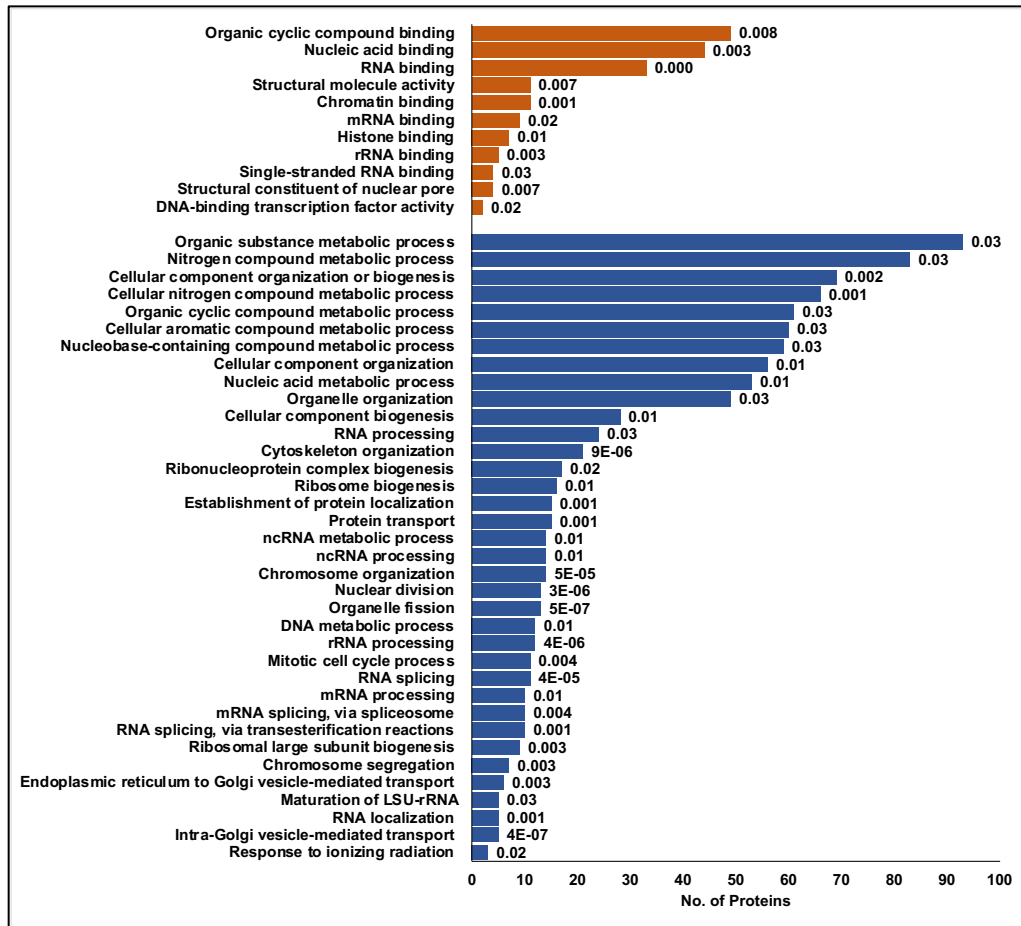

Supplement: Supplementary file 1 [file proteomes-13-00018-s001.zip › supplementary figure S1.pdf]
